# Tail-Aware Unsupervised Learning for Wideband Terahertz Quantized Beamforming in Sensor Data Transmission

**DOI:** 10.3390/s26217081

**Published:** 2026-11-09

**Authors:** Jiangnan Xie, Shujuan Yu, Yun Zhang, Liya Huang

**Affiliations:** 1 Bell Honors School, Nanjing University of Posts and Telecommunications, Nanjing 210003, China; q24010130@njupt.edu.cn; 2 College of Electronic and Optical Engineering & College of Flexible Electronics (Future Technology), Nanjing University of Posts and Telecommunications, Nanjing 210003, China; y021001@njupt.edu.cn (Y.Z.); huangly@njupt.edu.cn (L.H.)

**Keywords:** sensor data transmission, terahertz communications, analog beamforming, unsupervised learning, beam squint, imperfect channel state information

## Abstract

Data-intensive sensor networks require high-capacity wireless links to transmit sensor measurements. However, in wideband analog beamforming, beam squint reduces array gain, while imperfect channel state information (CSI) and finite-resolution phase shifters further degrade spectral efficiency (SE). To address these challenges, this paper proposes a center-anchored reliability-aware and tail-aware quantized beamforming (CRTQ-BF) algorithm for wideband terahertz sensor-data transmission over single-user multiple-input single-output (MISO) links with one radio-frequency (RF) chain. CRTQ-BF comprises three modules: the center-anchored reliability-aware refinement module learns a wideband phase correction from CSI reliability and a center-subcarrier phase reference; the tail-aware scenario learning module increases the weight of low-SE CSI-perturbation scenarios while retaining the mean-SE objective; and the quantization-aware phase projection module applies hard finite-phase quantization before scenario SE evaluation. Simulation results show that with increased inference time, CRTQ-BF achieves a higher mean SE with fewer trainable parameters than the physically anchored beamforming neural network (BFNN) baseline and demonstrates good robustness to CSI estimation errors. Remote-estimation simulations further show that CRTQ-BF improves sensor-data delivery reliability and reduces remote state-estimation error.

## 1. Introduction

As video surveillance and industrial monitoring applications expand, image and audio sensors are increasingly used in wireless multimedia sensor networks, generating data streams that require high-speed, reliable wireless transmission under bandwidth and transmit-power constraints [1,2]. To carry these data streams, measurements from local sensors are aggregated at a gateway and then transmitted over a terahertz link by its wireless transmitter. This transmitter uses beamforming techniques and antenna arrays to achieve array gain, thereby improving the received signal-to-noise ratio (SNR) and spectral efficiency (SE) for a given transmit power [3,4].

However, applying a shared analog beam over a wideband millimeter-wave or terahertz forwarding link introduces beam squint, which reduces array gain and SE, particularly near the band edges [5,6,7,8,9]. Beyond beam squint, imperfect channel state information (CSI) causes a mismatch between the designed beam and the actual channel, reducing the received SNR and SE [10]. Finite-resolution phase shifters restrict the phase of each antenna to a discrete level, introducing quantization errors and reducing the coherent combining gain across antennas [11,12]. Therefore, we investigate the optimization of mean and lower-tail SE in wideband terahertz analog beamforming under imperfect CSI and finite-resolution phase-shifter constraints with the aim of improving transmission performance on sensor-data links and remote state-estimation accuracy.

### 1.1. Prior Work

Chen et al. address beam squint through shared analog precoding and per-subcarrier digital processing [5]. In Refs. [13,14], shared analog precoders and per-subcarrier digital precoders are jointly optimized for wideband systems. In Ref. [15], frequency-selective hybrid precoding is studied under limited feedback. Huang et al. study adaptable hybrid beamforming with subset optimization for multi-user massive multiple-input multiple-output (MIMO) systems using multiple radio-frequency (RF) chains [16]. These architectures rely on multiple RF chains to provide multiple analog beams and digital-precoding degrees of freedom. However, the additional RF chains increase hardware cost, circuit power consumption, and calibration requirements.

Dai et al. introduce delay-phase precoding [17], while Chang and Chiueh combine low-resolution phase shifters with true-time-delay units [18]. These designs compensate for frequency-dependent beam misalignment at the cost of additional delay hardware and joint delay–phase control.

Learning-based methods connect neural networks with wireless resource optimization [19,20]. Lin and Zhu propose a beamforming neural network (BFNN) for narrowband single-RF-chain multiple-input single-output (MISO) beamforming and learn a constant-modulus beam from CSI [21]. When the same learned phase vector is applied over a wide band, the frequency-dependent array response moves the beam across subcarriers and can reduce the SE values near the band edges [5,6,22]. Kim et al. learn robust beams from estimated CSI and error statistics [23]. However, their beamforming weights require the continuous adjustment of amplitude and phase and cannot be directly implemented by constant-modulus, finite-resolution phase shifters. Ge and Dai improve multi-user MISO beamforming across different channel environments through domain adaptation [24]. Tarafder and Choi develop a deep-reinforcement-learning method for coordinated analog beamforming in millimeter-wave (mmWave) massive MIMO vehicular communication networks [3]. However, neither training formulation accounts for imperfect CSI. Errors in the input CSI can cause the predicted beams to be mismatched with the actual channel and reduce SE.

In Ref. [25], imperfect CSI and discrete phase shifts of a reconfigurable intelligent surface (RIS) are jointly considered through an expected weighted sum-rate objective. The deep quantization neural network (DQNN) uses a differentiable quantization approximation during training and discrete quantization during prediction; its improved variant (I-DQNN) adds a constraint to reduce the resulting performance gap. Xu et al. improve transmission robustness under bounded CSI errors through worst-case constraints [26]. Wang et al. impose outage constraints to limit the probability that the signal-to-interference-plus-noise ratio (SINR) falls below a prescribed threshold [27]. These methods have different limitations in protecting performance under unfavorable CSI errors: average-SE optimization can still yield low SE in some scenarios; worst-case guarantees depend on the prescribed error set; and outage-probability constraints do not guarantee a lower bound on SE under unfavorable CSI errors.

### 1.2. Contributions

Under these conditions, we propose center-anchored reliability-aware and tail-aware quantized beamforming (CRTQ-BF) for single-user wideband terahertz MISO sensor-data links with a single RF chain. The algorithm learns a shared finite-resolution analog beam from imperfect CSI through three integrated modules: the center-anchored reliability-aware refinement module (CARAR), the  tail-aware scenario learning module (TASL), and the quantization-aware phase projection module (QPP). The main contributions are as follows.

- Center-anchored reliability-aware refinement module: To adapt a center-subcarrier phase reference to the wideband channel while accounting for CSI uncertainty, we propose CARAR. The module retains the maximum-ratio transmission (MRT) phases of the center-subcarrier estimate, assigns equal amplitudes to all antennas, and combines the resulting reference with reliability-conditioned feature gating to learn a shared phase correction from frequency–antenna features.
- Tail-aware scenario learning module: To place greater emphasis on CSI-perturbation scenarios with low SE while retaining the mean-SE objective, we propose TASL. The module evaluates the same quantized beam across CSI-perturbation scenarios and combines their mean SE with the mean SE of the lowest-SE scenarios. The resulting loss directs network updates toward both average and lower-tail performance. At deployment, TASL further refines the QPP beam through one antenna-wise pass over adjacent discrete phases.
- Quantization-aware phase projection module: If TASL directly evaluates CSI-perturbation scenarios using a continuous-phase beam, phase quantization before deployment may change the scenario SE ranking, so the tail scenarios emphasized during training may differ from the lowest-SE scenarios of the deployable beam. To address this issue, QPP first applies hard phase quantization during training, and TASL uses the resulting shared beam to compute each scenario’s SE and select the lowest-SE scenarios. The tail objective thus evaluates a shared beam satisfying the finite-phase constraint. An identity straight-through estimator (STE) [28] passes the loss gradient to CARAR. We further derive the resulting scenario-weighted phase gradient and bounds on the phase and beam-vector errors introduced by projection.

Building on feature gating, risk-sensitive learning, and quantization-aware learning, these modules introduce innovative designs for wideband quantized shared beams. The feature-gating mechanism of Hu et al. recalibrates feature responses by learning channel interdependencies [29]; CARAR incorporates reliability information derived from estimated channel power and CSI-error variance into feature modulation across frequency–antenna positions, and it learns a shared wideband phase correction relative to the center-subcarrier reference. When negative SE is used as the loss, risk-sensitive learning based on conditional value-at-risk (CVaR) evaluates the risk of low-performance scenarios through a lower-tail mean SE [30]. Building on risk-sensitive learning, we introduce an innovative joint design of TASL and STE-based QPP that combines the lower-tail SE optimization of a wideband shared beam, finite-resolution phase constraints, and discrete phase refinement. This co-design further improves the spectral efficiency of wideband shared beams without increasing the number of trainable parameters.

In simulations with 64 transmit antennas and 32 subcarriers, the complete algorithm improves the mean SE by 13.40–21.55% over the anchored dense BFNN under 2-bit and 3-bit phase control at 10% and 20% fractional bandwidths, at 10 dB SNR and Gaussian CSI-error variance 0.1, with approximately 71% fewer trainable parameters. Further remote state-estimation simulations show that CRTQ-BF improves sensor-data delivery and reduces remote state-estimation error under imperfect CSI.

## 2. System Model

**Figure 1:**
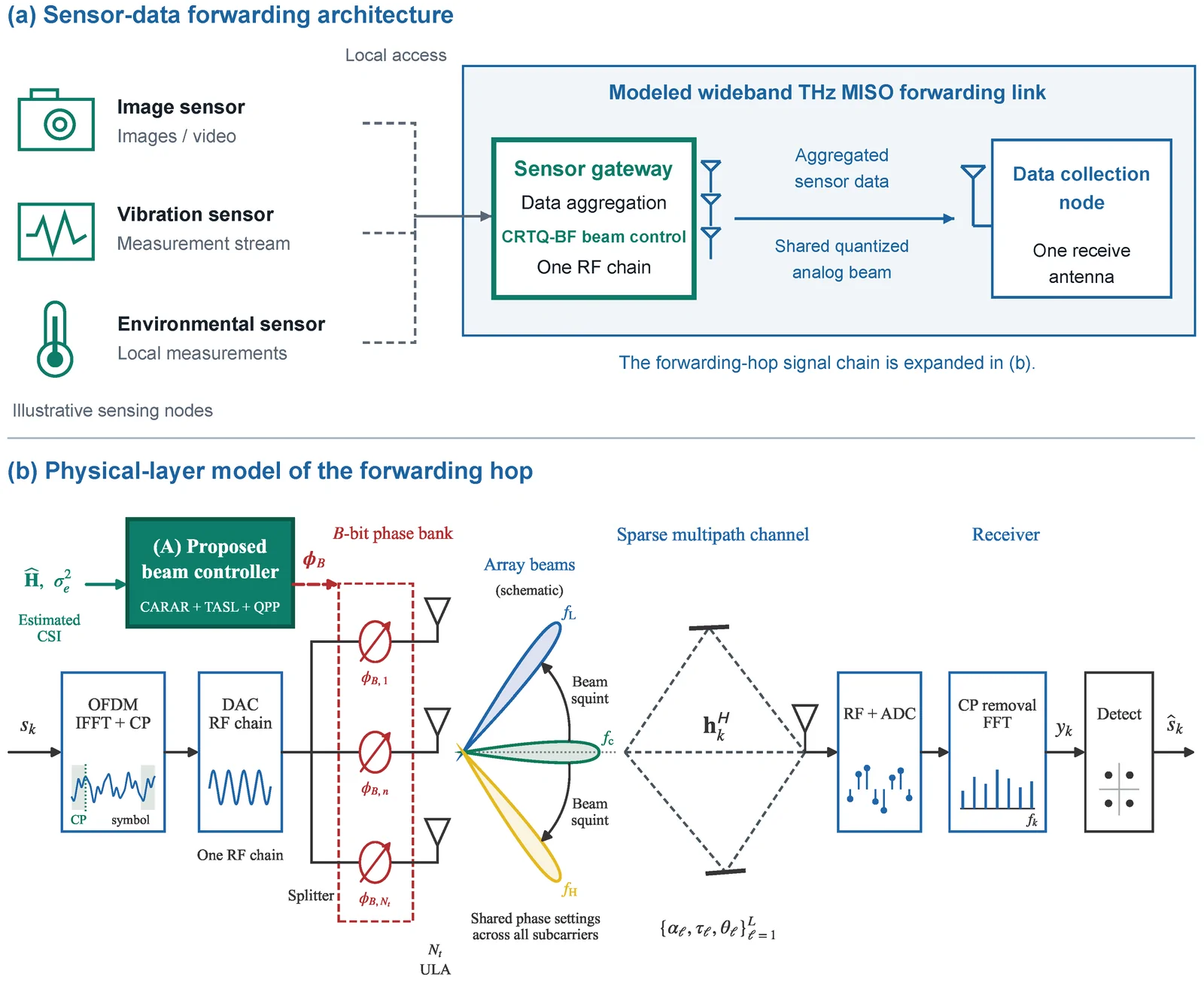
Sensor-data forwarding architecture and modeled physical-layer link. (**a**) Sensor nodes supply measurements to a gateway, which forwards the aggregated data to a collection node over a wideband terahertz link. (**b**) Single-RF-chain wideband terahertz MISO implementation of the forwarding hop. The CRTQ-BF controller uses estimated CSI and error variance to configure the *B*-bit phase shifters. The phase vector is shared across all *K* subcarriers ($f_{c}=300$ GHz, $N_{t}=64$). DAC and ADC denote digital-to-analog and analog-to-digital converters, respectively. IFFT denotes inverse fast Fourier transform, and CP denotes cyclic prefix.

Figure 1a illustrates the sensor-data forwarding architecture. Image, vibration, and environmental sensors are shown as representative data sources. A gateway aggregates their measurements and forwards the data to a collection node through its wireless transmit module. This module uses one RF chain, finite-resolution phase shifters, and an array of $N_{t}$ transmit antennas for terahertz transmission. With one receive antenna at the collection node, the forwarding hop forms a single-user wideband MISO link. The gateway maps the aggregated sensor data to transmitted symbols and uses the estimated forwarding-channel CSI to configure its beam. The collection node recovers the data stream for the subsequent processing of the sensor measurements.

Figure 1b shows the corresponding physical-layer signal chain. The green box highlights the proposed CRTQ-BF beam controller, which integrates CARAR, TASL, and QPP. Orthogonal frequency-division multiplexing (OFDM) carries data on *K* subcarriers. After OFDM modulation, the signal is upconverted to the terahertz carrier frequency and passes through the phase-shifter bank and antenna array for analog beamforming. All subcarriers share the same analog phase vector. The controller’s output $\phi_{B}$ controls the antenna phases, and the receiver uses a fast Fourier transform (FFT) to recover the subcarrier signals. Simulations evaluate the corresponding subcarrier-domain model. Here, $s_{k}$ denotes the transmitted data symbol on subcarrier *k*, with  $E[|s_{k}{|}^{2}]=1$, $y_{k}$ is the corresponding received symbol after the FFT, and ${\hat{s}}_{k}$ is its detected estimate.

### 2.1. Wideband Channel and Analog Beamforming

The subcarrier frequencies are denoted by $f_{k}=f_{c}+\delta f_{k}$, where $f_{c}$ is the carrier frequency; $f_{L}$ and $f_{H}$ in Figure 1 denote the frequencies of representative low- and high-frequency components. The subcarrier frequencies $f_{k}$ span the occupied frequency band, and $k_{0}$ denotes the selected central subcarrier used by the physical anchor. Following the wideband array formulation in [5,6], we use the frequency-dependent response of a uniform linear array (ULA) with antenna spacing *d*:

(1) $${\left[a(f,\theta )\right]}_{n}=\frac{1}{\sqrt{N_{t}}}e^{j2\pi fdn\sin\theta /c},\;n=0,\ldots ,N_{t}-1,$$

where *c* is the propagation speed and θ is the direction angle. The factor $1/\sqrt{N_{t}}$ normalizes the response so that ${∥a\left(f,\theta \right)∥}_{2}^{2}=1$. Its frequency dependence causes beam squint [5,6].

Following the geometric multipath representation in [5,31], we adopt the following simplified frequency-selective Saleh–Valenzuela-type sparse channel model:

(2) $$h_{k}=\sqrt{\frac{N_{t}}{L}}\sum_{\ell =1}^{L}\alpha_{\ell }e^{-j2\pi f_{k}\tau_{\ell }}a\left(f_{k},\theta_{\ell }\right),$$

where *L* is the number of paths, $\alpha_{\ell }$ and $\tau_{\ell }$ are the complex gain and delay of the *ℓ*-th path, $\theta_{\ell }$ is its departure angle, and $a(f_{k},\theta_{\ell })$ is the frequency-dependent array response. With independent $\alpha_{\ell }∼\mathrm{CN}\left(0,1\right)$, the prefactor gives $E[∥h_{k}{∥}_{2}^{2}]=N_{t}$, which is independent of *L*. Equivalently, the simulator draws $g_{\ell }∼\mathrm{CN}\left(0,1/L\right)$, absorbs the constant carrier-delay phase into $g_{\ell }$, and evaluates $\sqrt{N_{t}}\sum_{\ell }g_{\ell }e^{-j2\pi \delta f_{k}\tau_{\ell }}a\left(f_{k},\theta_{\ell }\right)$. All sampled frequencies share the same path gains, delays, and departure angles. The complex-Gaussian path gains define a normalized sparse fading environment for comparing shared beams.

Let v be the analog beamformer. Under phase-only analog control [11,21], it satisfies

(3) $$|v_{n}|=\frac{1}{\sqrt{N_{t}}},\;n=1,\ldots ,N_{t}.$$

For *B*-bit phase shifters, each phase belongs to a finite alphabet [11]:

(4) $$A_{B}=\left\{0,\frac{2\pi }{2^{B}},\ldots ,\frac{2\pi (2^{B}-1)}{2^{B}}\right\}.$$

In Figure 1, $\phi_{B}$ collects these quantized phase settings; $\phi_{B,n}$ controls antenna *n*. The three drawn branches represent antennas 1, *n*, and $N_{t}$. The SE averaged over subcarriers is [13,21]

(5) $$\;R\left(v\right)=\frac{1}{K}\sum_{k=1}^{K}\log_{2}\left(1+\rho |h_{k}^{H}{v|}^{2}\right).$$

Here, ρ denotes the per-subcarrier SNR in linear units. For an SNR of γ dB, $\rho =10^{\gamma /10}$.

### 2.2. Imperfect CSI and Shared-Beam Model

For the simulations in this study, we assume the following additive complex-Gaussian CSI-error model at the transmitter:

(6) $${\hat{h}}_{k}=h_{k}+e_{k},\;e_{k}∼\mathrm{CN}\left(0,\sigma_{e}^{2}I\right),$$

where $\sigma_{e}^{2}$ is the per-element error variance supplied to the controller. It uses the same normalization scale as the channel. The controller receives the estimated CSI and its associated error variance as inputs. The estimated CSI matrix in Figure 1 is $\hat{H}={\left[{\hat{h}}_{1},\ldots ,{\hat{h}}_{K}\right]}^{H}\in C^{K\times N_{t}}$: its *K* rows correspond to subcarriers and its $N_{t}$ columns to transmit antennas. A hat denotes an estimate, while ${\left(\cdot \right)}^{H}$ and ${\left(\cdot \right)}^{*}$ denote conjugate transpose and complex conjugation, respectively.

The subcarrier channels can be written in matrix form as follows:

(7) $$H=\left[\begin{array}{c} h_{1}^{H}\\ h_{2}^{H}\\ \vdots \\ h_{K}^{H} \end{array}\right]=\left[\begin{array}{ccc} h_{1,1}^{*} & \ldots & h_{1,N_{t}}^{*}\\ h_{2,1}^{*} & \ldots & h_{2,N_{t}}^{*}\\ \vdots & \ddots & \vdots \\ h_{K,1}^{*} & \ldots & h_{K,N_{t}}^{*} \end{array}\right]\in C^{K\times N_{t}}.$$

The estimated channel matrix $\hat{H}$ has entries ${\left[\hat{H}\right]}_{k,n}={\hat{h}}_{k,n}^{*}$. The network takes the real and imaginary parts of the estimated channel coefficients ${\hat{h}}_{k,n}$ as input. For unit-power independent subcarrier symbols $s={\left[s_{1},\ldots ,s_{K}\right]}^{T}$, the normalized received vector is

(8) $$\begin{array}{cc} z & =Hv,\\ y & =\sqrt{\rho }\mathrm{diag}\left(z\right)s+n,\;E\left[nn^{H}\right]=I_{K},\\ R(v) & =\frac{1}{K}1_{K}^{T}\log_{2}\left(1_{K}+\rho {\left|Hv\right|}^{⊙2}\right). \end{array}$$

Here, n is the normalized noise vector, $I_{K}$ is the K×K identity matrix, and $1_{K}$ is the length-*K* all-ones column vector. The operator diag(z) places the entries of z on the diagonal of a matrix. The superscript ${\left(\cdot \right)}^{T}$ denotes transpose. The operations ${\left|\cdot \right|}^{⊙2}$ and $\log_{2}\left(\cdot \right)$ denote elementwise squared magnitude and logarithm, respectively.

**Figure 2:**
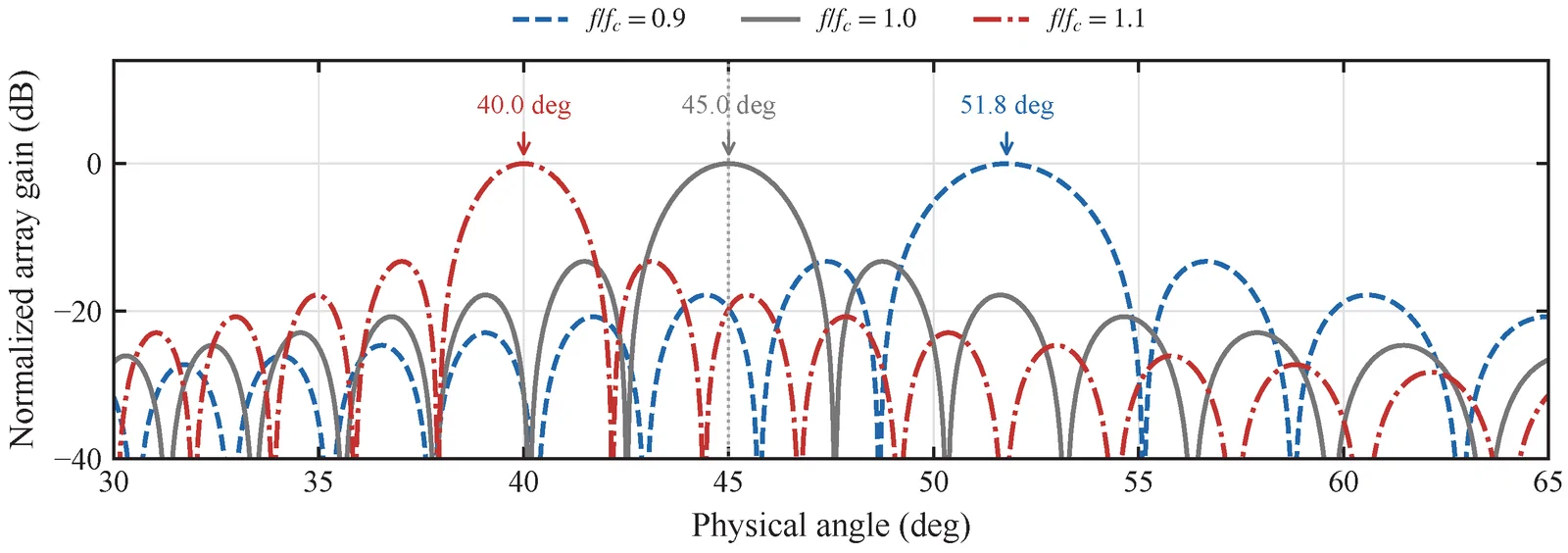
Normalized angular gain of a continuous-phase 64-element ULA steered to $45^{\circ }$ at $f_{c}$ for $f/f_{c}=0.9$, 1.0, and 1.1. Arrows mark main-lobe peaks; the dotted line marks $45^{\circ }$. Curves are calculated from (9).

For the single-path illustration in Figure 2, take $d=c/(2f_{c})$ and $v_{c}=a\left(f_{c},\theta_{0}\right)$. Here, $\theta_{0}$ is the reference beam direction. With $\xi =\left(f/f_{c}\right)\sin\theta -\sin\theta_{0}$, the normalized gain follows from (1):

(9) $$G\left(f,\theta \right)=|a^{H}\left(f,\theta \right)v_{c}{|}^{2}={\left|\frac{\sin(N_{t}\pi \xi /2)}{N_{t}\sin\left(\pi \xi /2\right)}\right|}^{2}.$$

The continuous extension gives G=1 at ξ=0. For  $|\left(f_{c}/f\right)\sin\theta_{0}|\leq 1$, the corresponding peak direction is $\theta_{\mathrm{peak}}\left(f\right)=\arcsin\left[\left(f_{c}/f\right)\sin\theta_{0}\right]$. In Figure 2, the receiver remains at $45^{\circ }$, while the off-center-frequency peaks shift away from this direction.

## 3. The Proposed CRTQ-BF Algorithm

In this section, we propose CRTQ-BF for wideband analog beamforming under imperfect CSI and finite phase resolution. The algorithm comprises three modules: center-anchored reliability-aware refinement (CARAR), tail-aware scenario learning (TASL), and quantization-aware phase projection (QPP).

### 3.1. Network Architecture

**Figure 3:**
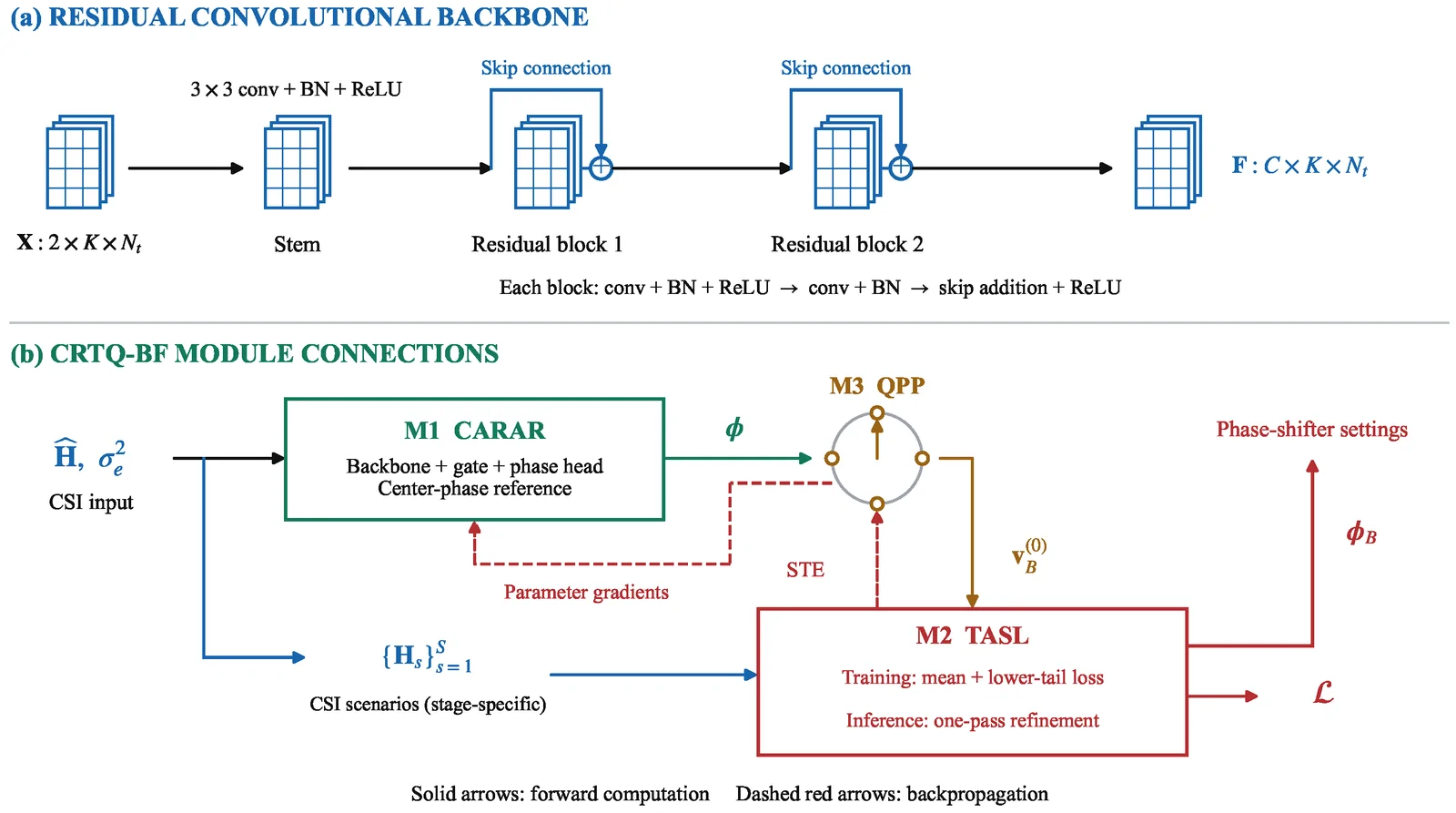
CRTQ-BF network overview. (**a**) Residual convolutional backbone within CARAR. (**b**) Module connections: CARAR generates continuous phases, QPP produces the initial discrete beam $v_{B}^{\left(0\right)}$, and TASL computes the training loss during training and produces the final phase-shifter settings $\phi_{B}$ at deployment. Dashed red arrows indicate backpropagation through QPP to CARAR. BN denotes batch normalization; the phase circle illustrates 2-bit projection.

The phase-prediction network uses a residual convolutional neural network (CNN) backbone [32], as shown in Figure 3a. The real and imaginary parts of the estimated channel coefficients ${\hat{h}}_{k,n}$ form the input $X\in R^{2\times K\times N_{t}}$. A 3×3 convolutional stem followed by batch normalization and a rectified linear unit (ReLU) maps the input to C=16 feature channels. Two residual blocks then extract frequency–antenna features $F=B_{\Theta }\left(X\right)\in R^{C\times K\times N_{t}}$. Here, $B_{\Theta }$ denotes the residual backbone, Θ collects the trainable network parameters, and $F_{c,k,n}$ denotes the feature at channel *c*, subcarrier *k*, and antenna *n*. Each block applies two 3×3 convolutions with batch normalization with ReLU after the first convolution and after the skip addition. Frequency averaging reduces the features to $C\times N_{t}$. A phase head with widths $CN_{t}\to 64\to N_{t}$ maps the pooled features to an $N_{t}$-dimensional phase correction.

Figure 3b illustrates the connections and data flow among the three modules. CARAR incorporates the backbone and phase head, inserts a CSI-reliability gate before frequency averaging, and adds the phase correction to a center-subcarrier reference. QPP maps the resulting continuous phases to the allowed phase levels and forms the shared beam $v_{B}$. During training, TASL evaluates this beam across CSI perturbations and combines the mean and lower-tail SE values into a loss. The loss gradient passes through QPP’s identity STE and updates the trainable layers within CARAR.

### 3.2. M1: Center-Anchored Reliability-Aware Refinement (CARAR)

**Figure 4:**
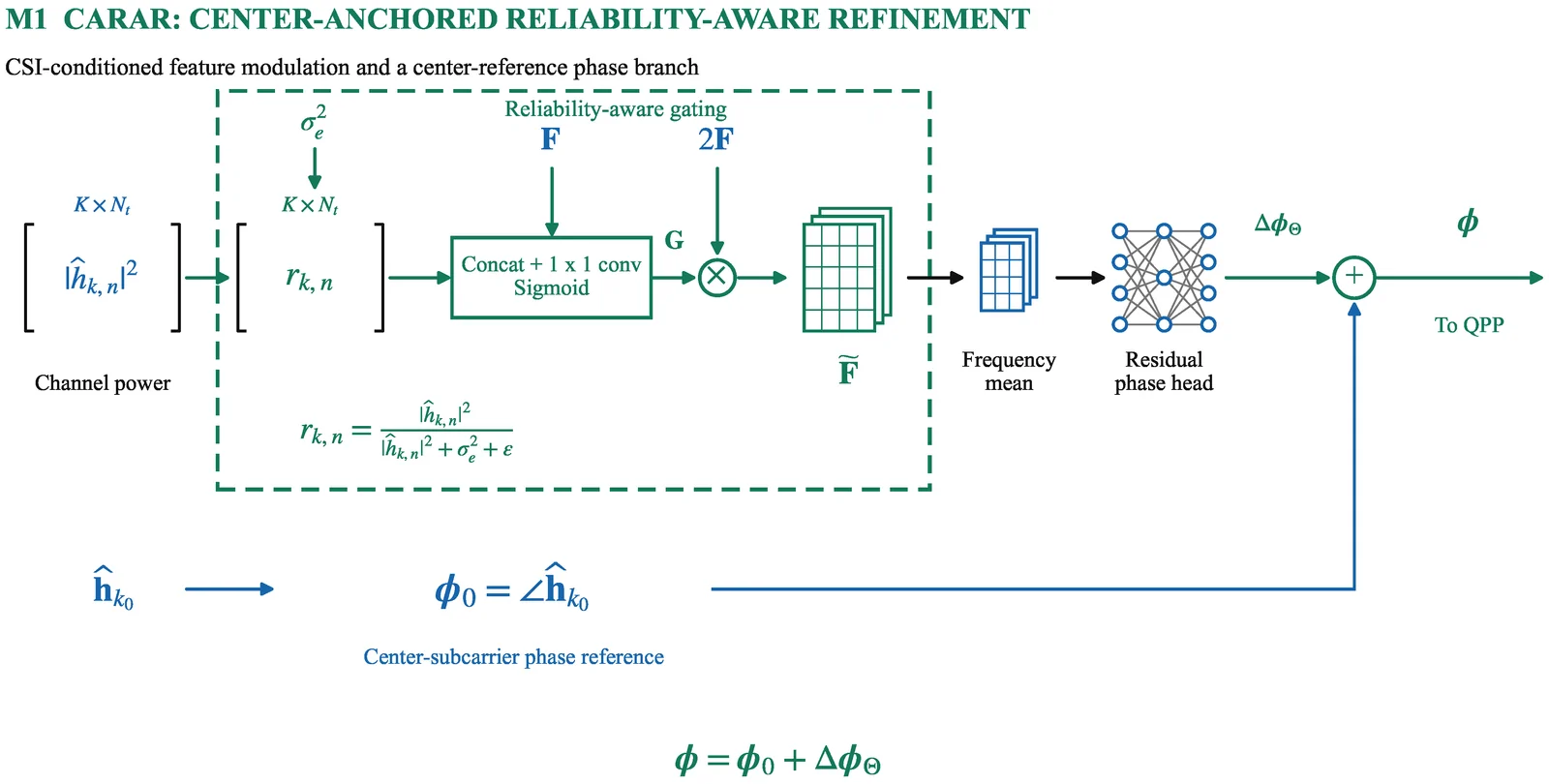
CARAR: reliability-conditioned feature modulation and center-reference phase refinement. CARAR’s residual backbone supplies F; the module outputs ϕ to QPP.

The center-subcarrier MRT phases provide a channel-dependent reference, but their alignment at the center tone leaves frequency-dependent misalignment across the band. Moreover, features extracted from an imperfect estimate have different levels of reliability. We propose CARAR to learn a wideband correction to this reference using features conditioned on the estimated channel power and error variance. The module combines a reference branch, reliability-conditioned gating, the residual convolutional backbone, and a phase-correction head. Following model-based learning [33], we take the MRT phases computed from estimated CSI as the network’s phase reference. Figure 4 expands the reliability gate and reference branch around the backbone features.

#### 3.2.1. Reliability Matrix and Feature Gating

CSI uncertainty affects the usefulness of the features extracted from the estimated channel. Feature recalibration modulates neural representations through learned weights [29]. The reliability gate within CARAR supplies the network with a local indicator of estimated channel power relative to the assumed error power, allowing feature modulation to adapt to the available CSI. The reliability matrix $r\in R^{K\times N_{t}}$ has entries

(10) $$r_{k,n}=\frac{|{\hat{h}}_{k,n}{|}^{2}}{|{\hat{h}}_{k,n}{|}^{2}+\sigma_{e}^{2}+\epsilon }.$$

The numerical stabilizer is $\epsilon =10^{-8}$. We concatenate the reliability matrix with the convolutional features and feed them into a pointwise gating layer. We initialize the gate as an identity mapping to preserve the backbone features at the start of training. The gate learns feature modulation weights from both the reliability matrix and the extracted channel features.

For the backbone features F introduced in Section 3.1, [F;r] denotes concatenation along the feature-channel axis: r forms one additional channel, giving a tensor of size $\left(C+1\right)\times K\times N_{t}$. The gate is

(11) $$\begin{array}{cc} G & =\mathrm{sigmoid}\left(W_{g}{*}_{1\times 1}\left[F;r\right]+b_{g}\right),\\ \tilde{F} & =F⊙\left[1+\beta (2G-1)\right],\;\beta =1. \end{array}$$

Here, G and $\tilde{F}$ are the gate weights and modulated features, which are both of size $C\times K\times N_{t}$. The operator ${*}_{1\times 1}$ applies a pointwise convolution with weight matrix $W_{g}\in R^{C\times (C+1)}$ and bias $b_{g}\in R^{C}$, which is shared across all (k,n) positions. The sigmoid $\mathrm{sigmoid}\left(x\right)={\left(1+e^{-x}\right)}^{-1}$ acts elementwise; ⊙ denotes elementwise multiplication, and 1 is an all-ones tensor of the same size as G. The modulation strength is fixed at β=1. Initializing $W_{g}=0$ and $b_{g}=0$ gives $G=\frac{1}{2}1$ and hence $\tilde{F}=F$. The modulation factor belongs to (0,2) for finite logits. Each entry $r_{k,n}$ of the reliability matrix is computed from the estimated CSI and supplied error variance and serves as a conditioning input to the gate. For a fixed channel estimate, its dependence on the error variance is

(12) $$\frac{\partial r_{k,n}}{\partial \sigma_{e}^{2}}=-\frac{|{\hat{h}}_{k,n}{|}^{2}}{{\left(|{\hat{h}}_{k,n}{|}^{2}+\sigma_{e}^{2}+\epsilon \right)}^{2}}\leq 0.$$

To analyze the gate’s sensitivity to its input features, we write (11) in matrix form. Let $M=KN_{t}$ and ${\left[F_{♭}\right]}_{c,j}=F_{c,k,n}$, where $j=\left(k-1\right)N_{t}+n$. Then,

(13) $$\begin{array}{cc} U & =\left[\begin{array}{ccc} F_{1,1,1} & \ldots & F_{1,K,N_{t}}\\ \vdots & \ddots & \vdots \\ F_{C,1,1} & \ldots & F_{C,K,N_{t}}\\ r_{1,1} & \ldots & r_{K,N_{t}} \end{array}\right],\\ G_{♭} & =\mathrm{sigmoid}\left(\left[\begin{array}{cc} W_{F} & w_{r} \end{array}\right]U+b_{g}1_{M}^{T}\right),\\ {\tilde{F}}_{♭} & =2F_{♭}⊙G_{♭}. \end{array}$$

The dimensions are $U\in R^{(C+1)\times M}$, $W_{F}\in R^{C\times C}$, and  $w_{r},b_{g}\in R^{C}$ with $W_{g}=\left[W_{F}\;w_{r}\right]$. The subscript ♭ indicates the same reshaping for the gate weights and modulated features; $1_{M}$ is the length-*M* all-ones column vector.

The gate has a local sensitivity matrix. Define $f=\mathrm{vec}(F_{♭})$, $g=\mathrm{vec}(G_{♭})$, $D_{f}=\mathrm{diag}\left(f\right)$, and  $D_{g}=\mathrm{diag}\left(g⊙\left(1-g\right)\right)$. Here, vec stacks matrix columns into a column vector, and the unsubscripted 1 has length CM. Holding the reliability matrix fixed gives

(14) $$\frac{\partial \mathrm{vec}({\tilde{F}}_{♭})}{\partial f^{T}}=2\mathrm{diag}\left(g\right)+2D_{f}D_{g}\left(I_{M}⊗W_{F}\right).$$

Here, ⊗ denotes the Kronecker product and $I_{M}$ is the M×M identity matrix. Equation (14) describes how the gate output responds to changes in its input features with the reliability matrix fixed. At initialization, the Jacobian equals the CM×CM identity matrix $I_{CM}$, so gradients pass through the gate unchanged.

The pointwise gating layer has C(C+1) weights and *C* biases, totaling C(C+2)=288 trainable parameters for C=16. Its multiply–accumulate count is $KN_{t}C\left(C+1\right)$, which grows linearly with the number of frequency–antenna positions for fixed *C*.

#### 3.2.2. Reference Phase and Residual Correction

The center-subcarrier reference formed by retaining the MRT phases and assigning equal amplitudes to all antennas is

(15) $$v_{0}=\frac{1}{\sqrt{N_{t}}}e^{j∠{\hat{h}}_{k_{0}}}.$$

For even *K*, we select the higher-frequency subcarrier of the two nearest to $f_{c}$ as the reference with $k_{0}=K/2+1$. This constant-modulus vector uses the MRT phase alignment with equal antenna amplitudes. We refer to it as the center-subcarrier phase reference below.

After gating, frequency pooling and the phase head give

(16) $$\begin{array}{cc} P_{c,n} & =\frac{1}{K}\sum_{k=1}^{K}{\tilde{F}}_{c,k,n},\\ \Delta \phi_{\Theta } & =W_{2}\mathrm{ReLU}\left(W_{1}\mathrm{vec}\left(P^{T}\right)+b_{1}\right)+b_{2}. \end{array}$$

Here, $P\in R^{C\times N_{t}}$ contains the frequency-averaged features; $W_{1},W_{2}$ and $b_{1},b_{2}$ are the phase head’s weights and biases. The vector $\mathrm{vec}(P^{T})$ lists antennas within each feature channel. For C=16 and $N_{t}=64$, $W_{1}\in R^{64\times 1024}$ and $W_{2}\in R^{64\times 64}$. The phase head produces $N_{t}$ phase corrections, which are added to the reference phases:

(17) $$\phi =∠{\hat{h}}_{k_{0}}+\Delta \phi_{\Theta }\left(\hat{H},\sigma_{e}^{2}\right).$$

The residual head is zero-initialized, so the untrained continuous-phase model reduces exactly to the physical anchor. With finite-bit deployment, the  corresponding initial beam uses the quantized anchor phases $Q_{B}\left(∠{\hat{h}}_{k_{0}}\right)$. CARAR receives $\left(\hat{H},\sigma_{e}^{2}\right)$, adds the learned correction $\Delta \phi_{\Theta }$ to the reference phase, and  passes the corrected phase vector ϕ to QPP, as shown in Figure 4.

### 3.3. M2: Tail-Aware Scenario Learning (TASL)

**Figure 5:**
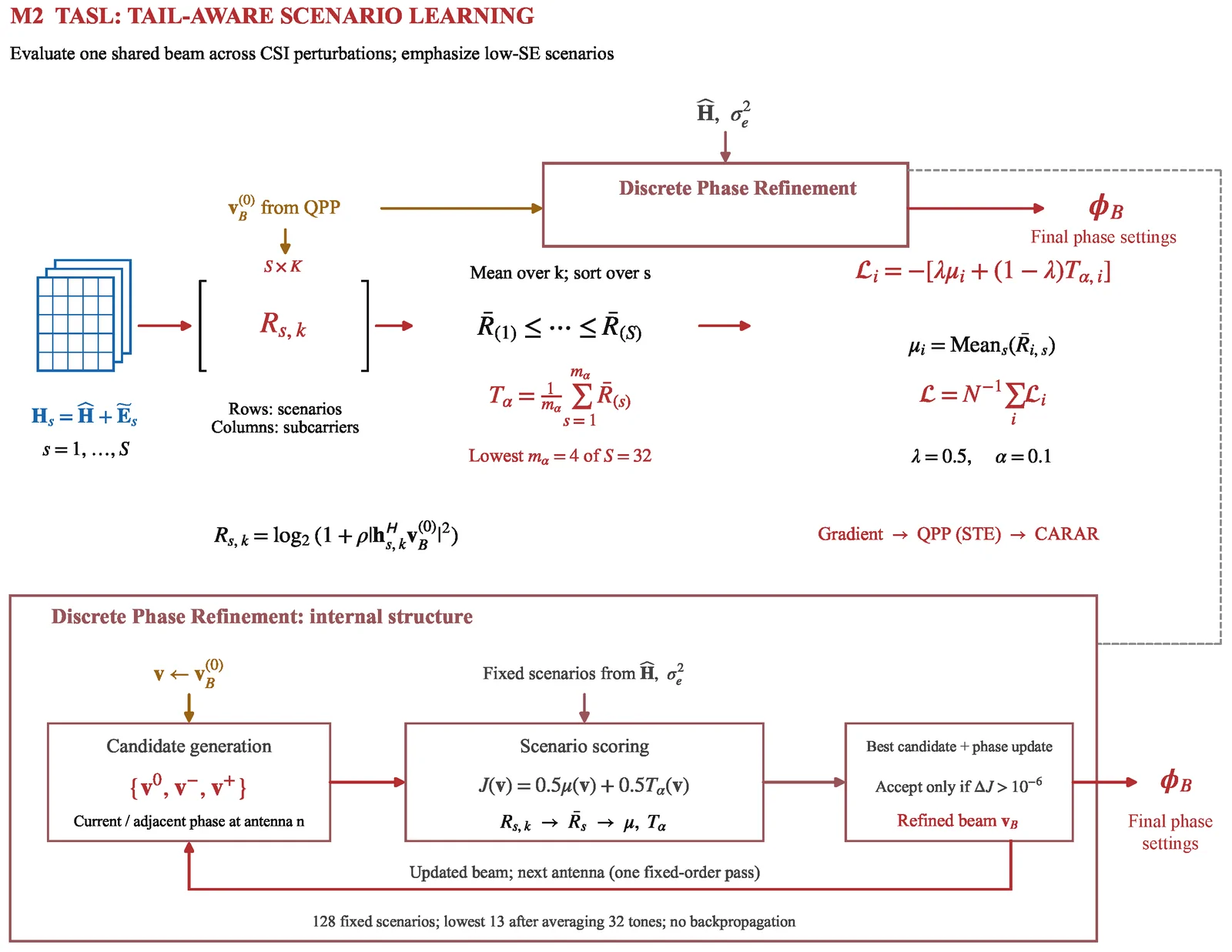
TASL: shared-beam evaluation across CSI scenarios and mean/tail loss calculation with phase refinement at deployment. During deployment, TASL receives $v_{B}^{\left(0\right)}$ and outputs final phase settings $\phi_{B}$. $T_{\alpha }$ denotes the lower-tail mean; *N* is the batch size.

CARAR incorporates CSI reliability into phase generation. The training objective must also specify how performance under different channel perturbations influences the learned phases. Averaging the scenario SE values gives each scenario the same direct weight. We propose TASL to give the lowest-SE scenarios additional weight while retaining the average-SE term. For the current M1 output, QPP supplies the finite-phase beam $v_{B}$ used in every scenario, as shown in Figure 5. During training, we generate the scenarios around each estimated channel:

(18) $$h_{s,k}={\hat{h}}_{k}+{\tilde{e}}_{s,k},\;{\tilde{e}}_{s,k}∼\mathrm{CN}\left(0,\sigma_{e}^{2}I\right).$$

We stack $h_{s,k}^{H}$ as the rows of $H_{s}$, following (7). The perturbations specify the Gaussian uncertainty model used for training, and their variance controls the spread around the estimate. One beam $v_{B}$ is computed from the estimate and evaluated across its *S* scenarios. Held-out physical channels are used for the final performance evaluation.

For scenario *s*, the SE averaged over subcarriers is

(19) $${\bar{R}}_{s}=\frac{1}{K}\sum_{k=1}^{K}\log_{2}\left(1+\rho |h_{s,k}^{H}v_{B}{|}^{2}\right).$$

For *S* scenarios, let $m_{\alpha }=\max\left(1,\left\lceil \alpha S\right\rceil \right)$ and let $S_{\alpha }$ contain the indices of the $m_{\alpha }$ smallest values among ${\left\{{\bar{R}}_{s}\right\}}_{s=1}^{S}$. The empirical lower-tail mean is

(20) $${\mathrm{Tail}}_{\alpha }\left(\left\{{\bar{R}}_{s}\right\}\right)=\frac{1}{m_{\alpha }}\sum_{s\in S_{\alpha }}{\bar{R}}_{s}.$$

Following the loss-based CVaR formulation [30,34], taking $-{\bar{R}}_{s}$ as the loss makes the lower-tail SE mean in (20) equal to the negative empirical CVaR of that loss at tail probability $m_{\alpha }/S$. TASL incorporates this term to improve SE in the lowest-SE CSI-perturbation scenarios. The training objective is

(21) $$L=-\frac{1}{N}\sum_{i=1}^{N}\left[\lambda {\mathrm{Mean}}_{s}\left({\bar{R}}_{i,s}\right)+\left(1-\lambda \right){\mathrm{Tail}}_{\alpha }\left(\left\{{\bar{R}}_{i,s}\right\}\right)\right].$$

The two terms balance the mean SE and lower-tail SE across sampled CSI-perturbation scenarios. Here, *N* is the mini-batch size, ${\bar{R}}_{i,s}$ is the wideband mean SE of training sample *i* in perturbation scenario *s*, and  ${\mathrm{Mean}}_{s}\left({\bar{R}}_{i,s}\right)=S^{-1}\sum_{s=1}^{S}{\bar{R}}_{i,s}$. The training coefficient λ weights the scenario mean. We set α=0.1 and λ=0.5 and evaluate true-channel SE values separately on held-out channels. We choose these values empirically: α=0.1 targets approximately the lowest 10% of scenario SE values, and λ=0.5 balances the mean SE and lower-tail SE in the training objective.

For a fixed training example, away from ties at the tail boundary, the  objective assigns each scenario the weight

(22) $$w_{s}=\frac{\lambda }{S}+\frac{1-\lambda }{m_{\alpha }}I\left\{s\in S_{\alpha }\right\}.$$

Here, I{·} equals one when the condition holds and zero otherwise. These nonnegative weights sum to one. With S=32, α=0.1, and λ=0.5, the four scenarios with the lowest SE receive weight 9/64 each, and the other scenarios receive 1/64 each.

The complete scenario evaluation can also be written as

(23) $$\begin{array}{cc} H & =\left[\begin{array}{c} H_{1}\\ H_{2}\\ \vdots \\ H_{S} \end{array}\right]\in C^{SK\times N_{t}},\\ Z & =\left[\begin{array}{ccc} h_{1,1}^{H}v_{B} & \ldots & h_{1,K}^{H}v_{B}\\ \vdots & \ddots & \vdots \\ h_{S,1}^{H}v_{B} & \ldots & h_{S,K}^{H}v_{B} \end{array}\right],\\ r_{\mathrm{sc}} & =\frac{1}{K}\log_{2}(1_{S\times K}+{\rho |Z|}^{⊙2})1_{K}. \end{array}$$

Here, $\mathrm{vec}\left(Z^{T}\right)=Hv_{B}$. Each row represents one scenario, and each column represents one subcarrier. Frequency averaging first produces the *S* entries of $r_{\mathrm{sc}}$; tail selection then operates on these scenario SE values. Hence, the low-SE emphasis concerns CSI uncertainty after wideband averaging.

Let $t\in {\left\{0,1\right\}}^{S}$ identify the selected scenarios with $1_{S}^{T}t=m_{\alpha }$. The utility and the diagonal factors needed for its phase gradient are

(24) $$\begin{array}{cc} w & =\frac{\lambda }{S}1_{S}+\frac{1-\lambda }{m_{\alpha }}t,\;J=w^{T}r_{\mathrm{sc}},\\ D_{(s-1)K+k,(s-1)K+k} & =\frac{w_{s}}{1+\rho |Z_{s,k}{|}^{2}}. \end{array}$$

The diagonal matrix $D\in R^{SK\times SK}$ couples the scenario emphasis to the SE sensitivity at each subcarrier.

During deployment, TASL starts from the QPP quantized beam $v_{B}^{\left(0\right)}$ and its phases $\phi_{B}^{\left(0\right)}$, and it refines them to obtain the final beam $v_{B}^{★}$ and phase settings $\phi_{B}^{★}$. TASL uses $S_{r}=128$ fixed Gaussian selection scenarios from (18), scoring candidate phases at a design SNR of $\rho_{d}$:

(25) $$\begin{array}{cc} {\bar{R}}_{s}^{r}\left(v\right) & =\frac{1}{K}\sum_{k=1}^{K}\log_{2}\;\left(1+\rho_{d}{\left|h_{s,k}^{H}v\right|}^{2}\right),\\ m_{r} & =\max(1,\left\lceil 0.1S_{r}\right\rceil )=13,\\ J_{\beta_{r}}\left(v\right) & =\left(1-\beta_{r}\right)\frac{1}{S_{r}}\sum_{s=1}^{S_{r}}{\bar{R}}_{s}^{r}\left(v\right)+\frac{\beta_{r}}{m_{r}}\sum_{s\in S_{r}\left(v\right)}{\bar{R}}_{s}^{r}\left(v\right),\;\beta_{r}=0.5. \end{array}$$

Here, ${\bar{R}}_{s}^{r}\left(v\right)$ is the wideband mean SE of candidate beam v in perturbation scenario *s*, and $m_{r}$ is the number of scenarios selected to compute the lower-tail mean. The score $J_{\beta_{r}}\left(v\right)$ is a weighted combination of the mean SE over all scenarios and the lower-tail mean SE, which is used to compare candidate beams. The set $S_{r}\left(v\right)$ contains the indices of the $m_{r}$ scenarios with the lowest wideband mean SE for candidate beam v, and $\beta_{r}=0.5$ is the weight of the lower-tail mean.

TASL performs one phase-refinement pass in antenna-index order $1,\ldots ,N_{t}$, comparing the current phase and its two adjacent discrete phases and updating the beam according to their scores. The discrete phase set is treated cyclically when selecting adjacent phases. With the other antenna phases held fixed, TASL evaluates the three candidate beams using the same CSI perturbation scenarios. For each beam, it selects the 13 scenarios with the lowest wideband mean SE to compute the lower-tail mean.

To reduce the cost of evaluating candidate beams, TASL stores the effective channel response $z_{s,k}=h_{s,k}^{H}v$ for the current beam. When the beamforming weight of antenna *n* changes from $v_{n}$ to $v_{n}^{\prime }$, the response is updated as follows:

(26) $$z_{s,k}^{\prime }=z_{s,k}+h_{s,k,n}^{*}\left(v_{n}^{\prime }-v_{n}\right).$$

Here, $h_{s,k,n}$ is the *n*th element of the channel vector $h_{s,k}$. TASL updates the corresponding effective channel responses while updating the phases. The final phase vector $\phi_{B}^{★}$ defines the beam

(27) $$v_{B}^{★}=N_{t}^{-1/2}\exp\left(j\phi_{B}^{★}\right).$$

### 3.4. M3: Quantization-Aware Phase Projection (QPP)

**Figure 6:**
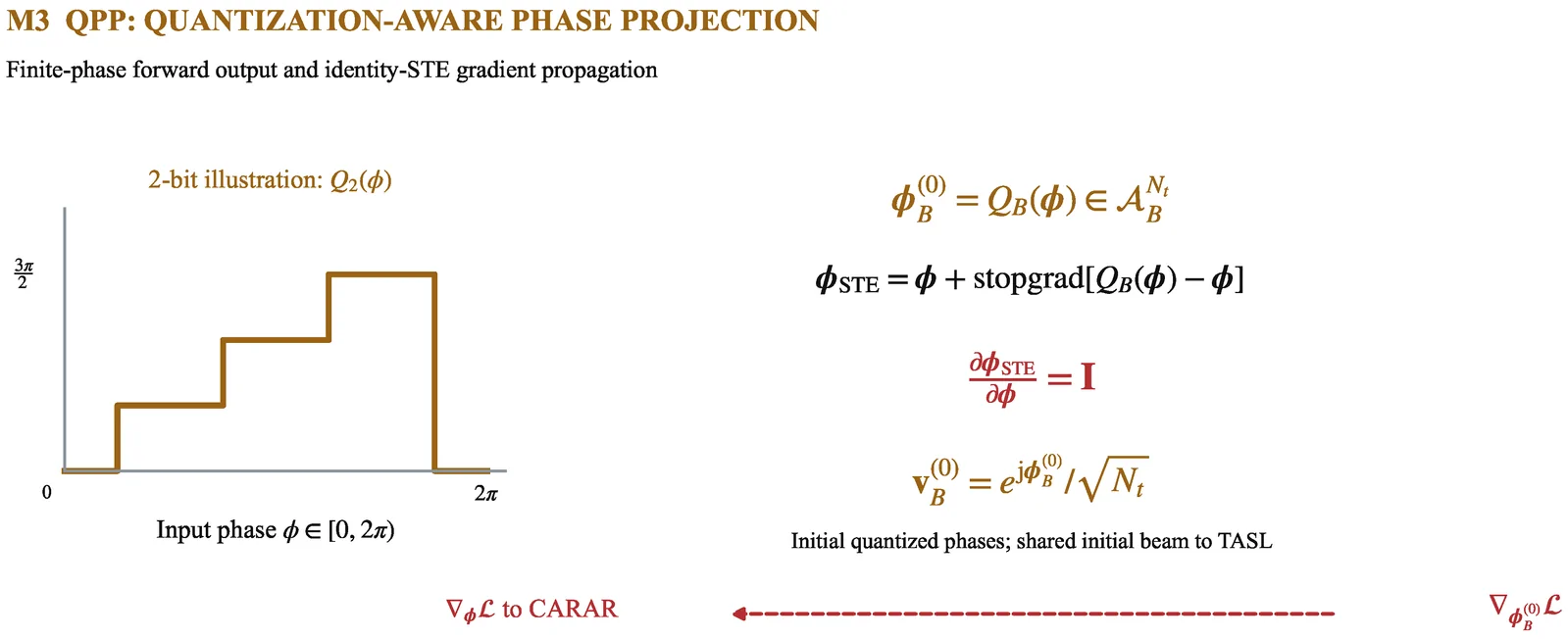
QPP: hard finite-phase forward projection and identity-STE backward path. The projected beam initializes TASL phase refinement at deployment.

TASL evaluates a beam that must be realizable by a *B*-bit phase bank. Hard rounding has zero derivative inside each phase bin and is discontinuous at the boundaries. In CRTQ-BF, QPP places the hard phase projection before scenario-SE evaluation, so the quantized shared beam determines both the SE values and the lower-tail scenario selection. QPP combines this deployment-consistent forward path with an identity straight-through gradient in the backward pass. QPP receives the phase vector from CARAR and returns the initial quantized settings $\phi_{B}$ and the shared beam $v_{B}$ used by TASL. QPP includes the discrete settings in every training SE evaluation, so the learned correction is optimized with the same phase alphabet used at deployment. Figure 6 shows the phase mapping and the identity-STE gradient path. First, wrap each phase onto [0,2π) and project it onto the nearest allowed phase:

(28) $$\begin{array}{cc} \phi^{\circ } & =\mathrm{mod}(\phi ,2\pi ),\\ Q_{B}\left(\phi \right) & =\frac{2\pi }{2^{B}}\mathrm{mod}\left(\mathrm{round}\left(\frac{2^{B}\phi^{\circ }}{2\pi }\right),2^{B}\right), \end{array}$$

which maps every phase to $A_{B}$ in the forward pass.

Converting the quantized phases to a unit-norm beam gives

(29) $$v_{B}=\frac{1}{\sqrt{N_{t}}}e^{j\phi_{B}},\;\phi_{B}=Q_{B}\left(\phi \right),$$

which satisfies the constant-modulus and finite-alphabet constraints in the forward pass.

Hard quantization has zero derivative inside each phase bin and is discontinuous at its boundaries. Using the straight-through gradient-estimation approach in [28], we train the preceding network using the following identity STE:

(30) $$\begin{array}{cc} \phi_{B} & =\phi +\mathrm{sg}\left[Q_{B}\left(\phi \right)-\phi \right],\\ {\left.\frac{\partial \phi_{B}}{\partial \phi^{T}}\right|}_{\mathrm{STE}} & =I_{N_{t}}. \end{array}$$

Here, sg preserves its argument in the forward pass and assigns it zero derivative in the backward pass. Thus, the forward phases belong exactly to $A_{B}$, while the backward computation uses a surrogate identity Jacobian. Gradients continue through phase addition, the phase head, frequency averaging, and the reliability gate within CARAR to the residual backbone. The optimizer updates the trainable weights and biases; the prescribed phase alphabet remains fixed.

With $z_{s,k}=h_{s,k}^{H}v_{B}$, the identity STE gives the surrogate SE gradient

(31) ∂R¯s∂ϕn≃−2ρKln2∑k=1KIm{zs,k∗hs,k,n∗vB,n}1+ρ|zs,k|2.

This surrogate carries the SE gradient through the quantized phase layer to the preceding network.

For the quantized beam $v_{B}=v_{B}^{\left(0\right)}$ produced by QPP, the phase error and beam distance can be bounded as follows. Let $\tilde{v}$ denote the continuous beam before projection and define the circular quantization error $\varepsilon_{n}={\mathrm{wrap}}_{\left[-\pi ,\pi \right)}\left(Q_{B}\left(\phi_{n}\right)-\phi_{n}\right)$. Mapping each phase to the nearest quantized level gives

(32) $$\begin{array}{cc} |\varepsilon_{n}| & \leq \frac{\pi }{2^{B}},\;\left|v_{B,n}\right|=\frac{1}{\sqrt{N_{t}}},\\ ∠v_{B,n} & \in A_{B}\;\left(\mathrm{mod}\;2\pi \right). \end{array}$$

Using $|e^{j\varepsilon }-1|=2|\sin\left(\varepsilon /2\right)|$ yields

(33) $$\begin{array}{cc} ∥v_{B}-\tilde{v}{∥}_{2}^{2} & =\frac{4}{N_{t}}\sum_{n=1}^{N_{t}}\sin^{2}\left(\varepsilon_{n}/2\right)\\ & \leq 4\sin^{2}\left(\frac{\pi }{2^{B+1}}\right). \end{array}$$

The bound decreases as phase resolution increases and characterizes the beam-vector discrepancy introduced by projection.

#### Coupled Training Gradient

Combining the TASL weights in (24) with the QPP surrogate in (31) yields the compact phase-gradient expression

(34) Aw=HHDH⪰0,∇ϕJ≃2ρKln2Imdiag(vB∗)AwvB.

Here, $A_{w}$ is an instantaneous weighted channel Gram matrix whose diagonal weights depend on the current beam and scenario ordering. A strict gap at the tail-selection boundary fixes the selected set locally. At a boundary tie, the selected SE weights are a supergradient of the concave utility; their negatives are a subgradient of the loss (the negative utility) in SE coordinates. Backpropagation selects an active branch. The phase expression gives the exact continuous-phase derivative when no quantizer is present and a straight-through surrogate for finite-bit forward evaluation.

If $J_{\Theta }=\partial \phi /\partial \Theta^{T}$ denotes the real phase-network Jacobian, the per-example loss gradient is

(35) ∇ΘLi≃−JΘT2ρKln2Imdiag(vB∗)AwvB.

Equations (14) and (35) show the three roles in the parameter gradient: the gate changes the network Jacobian, the tail objective changes D, and phase projection determines the feasible forward beam $v_{B}$.

## 4. Simulation Results

This section defines the evaluation metrics and experimental settings; then, it examines transmission performance, robustness, phase resolution, computational complexity, and remote state estimation.

### 4.1. Evaluation Metrics

We compare the transmission performance of CRTQ-BF and the other algorithms using SE, which is calculated by (5) on held-out true channels. SE measures the achievable information rate per unit bandwidth, which is in bit/s/Hz. Performance analysis uses the test-channel mean SE and its 10th percentile (P10) to assess average performance and performance among low-SE channels, respectively. For each independent run, let $R_{\left(1\right)}\leq \cdots \leq R_{\left(M\right)}$ denote the ordered SE values of the *M* true test-channel samples. The cross-channel 10th percentile is

(36) $$P_{10}=\left(1-\delta \right)R_{\left(j\right)}+\delta R_{\left(j+1\right)},$$

where h=1+0.1(M−1), j=⌊h⌋, and δ=h−j.

Robustness analysis examines changes in the mean SE as the CSI error, supplied error variance, and bandwidth vary. The scenario lower-tail SE in (20) additionally measures performance in low-SE perturbation scenarios for each channel estimate. Training convergence is assessed using the trajectories of each method’s validation objective; the training loss is defined in (21).

Complexity analysis uses trainable parameter count and model-state storage in bytes to measure model size together with the core multiply–accumulate (MAC) count, central processing unit (CPU) network inference time per sample, and complete deployment-call time on a graphics processing unit (GPU) to measure computational cost. Phase-resolution analysis uses the mean SE.

Sensor-data forwarding is assessed by the frame delivery success rate, which is the fraction of frames received within their transmission intervals. Remote estimation uses mean conditional error variance and empirical mean-squared error (MSE), which is the squared difference between the monitored state and its remote estimate averaged over evaluated updates and trajectories. Lower errors indicate more accurate estimation.

### 4.2. Simulation and Training Settings

**Figure 7:**
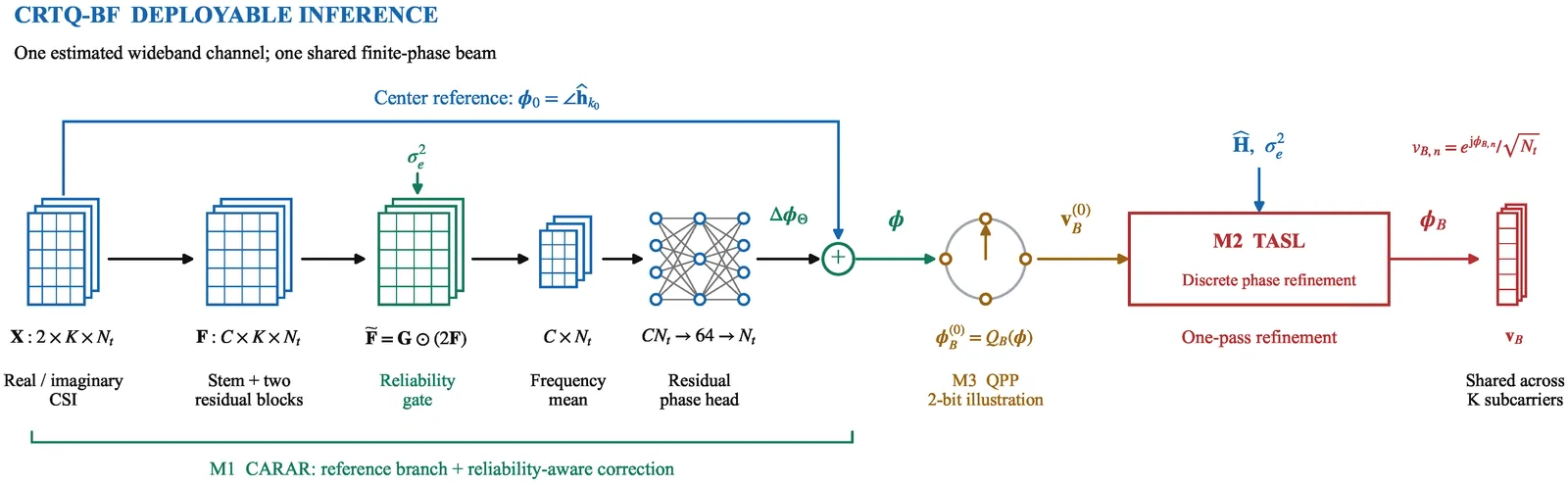
CRTQ-BF deployment path. The controller maps estimated wideband CSI and error variance to one shared finite-resolution phase vector, which configures the phase-shifter bank in the single-RF-chain signal path ($N_{t}=64$, K=32).

During training, TASL evaluates the quantized beam across CSI-perturbation scenarios and sends the loss gradient through QPP’s identity STE to update CARAR. During deployment, CARAR and QPP map the estimated CSI and supplied error variance to an initial quantized beam, which TASL refines to obtain the phase-shifter settings, as shown in Figure 7.

The end-to-end algorithm is summarized in Algorithm 1. The network configurations used in the controlled comparisons are listed in Table 1.

We consider a 64-element uniform linear array and 32 subcarriers at a carrier frequency of 300 GHz with antenna spacing $d=c/(2f_{c})$. These settings describe the gateway transmitter and its forwarding link in Figure 1; the receive antenna belongs to the data collection node. Table 2 summarizes the three-path simulation setup.

We repeat the experiments with five random seeds, each using 8192 training, 1024 validation, and 2048 test channels. Each metric is computed separately for each run, and its mean and sample standard deviation are then calculated across random seeds. For methods using scenario-based training, each training example uses 32 estimate-based perturbation scenarios. The nominal evaluation SNR is 10 dB. Path angles and delays are independently drawn from U[−π/2,π/2] and U[0,5ns], respectively. The *K* frequencies are uniformly spaced across the stated bandwidth, including both endpoints. At a 300 GHz carrier and 20% fractional bandwidth, adjacent frequency samples are approximately 1.94 GHz apart. These samples represent the channel response across the full bandwidth for shared-beam design and SE evaluation. Within the prescribed path-delay range, the relative phases of the multipath components can differ substantially between adjacent frequency samples, potentially resulting in weak channel correlation between these samples. Training uses Adam with $\left(\beta_{1},\beta_{2}\right)=\left(0.9,0.999\right)$, a numerical stabilizer of $10^{-8}$, no weight decay, and gradient-norm clipping at 5. The perturbation scenarios are resampled each epoch; model selection and stopping use the objective evaluated on a separate validation set.
**Algorithm 1** CRTQ-BF training and deployment algorithm.**Input:** Estimated wideband CSI $\hat{H}$, CSI error variance $\sigma_{e}^{2}$, phase resolution *B*, and network parameters.**Training scenarios:** CSI-uncertainty scenarios ${\left\{H_{s}\right\}}_{s=1}^{S}$. Compute the center-subcarrier phase $∠{\hat{h}}_{k_{0}}$ and use it as the physical MRT anchor. Form the reliability matrix $r_{k,n}=|{\hat{h}}_{k,n}{|}^{2}/\left(\right|{\hat{h}}_{k,n}{|}^{2}+\sigma_{e}^{2}+\epsilon )$. Extract frequency–antenna features with the residual convolutional backbone and apply reliability-conditioned feature gating. Predict the residual phase $\Delta \phi_{\Theta }$ and form $\phi =∠{\hat{h}}_{k_{0}}+\Delta \phi_{\Theta }$. Project the phase to the exact finite alphabet using $Q_{B}\left(\phi \right)$ and form the constant-modulus beam $v_{B}$. **Training only:** Evaluate the shared beam over CSI-uncertainty scenarios, compute the mean SE and empirical lower-tail SE, and update the network by minimizing (21), using the identity STE through phase projection. **Deployment:** Generate Gaussian perturbation scenarios conditioned on estimated CSI and the supplied error variance. TASL refines the quantized beam produced by QPP in one pass over the antennas, using a joint score based on mean and lower-tail SE.**Output:** Final finite-resolution analog beamformer $v_{B}^{★}$ and phase-shifter settings $\phi_{B}^{★}$.

### 4.3. Performance Analysis

Table 3 compares SE under 2-bit and 3-bit phase control at fractional bandwidths of 10% and 20%.

To further compare CRTQ-BF with classical beamforming methods, we include two baselines: the wideband analog-beam optimization of Sohrabi and Yu [13] and the orthogonal matching pursuit (OMP) method of El Ayach et al. [35]. In the single-RF-chain setting, the former constructs its objective from the mean estimated-channel covariance across subcarriers and updates the discrete phases coordinate-wise. The latter selects a beam from a fixed dictionary of 256 array-response directions using the estimated center-subcarrier channel. In addition, CARAR+QPP uses the mean SE under estimated CSI as its training and validation objective.

Under 2-bit control, CRTQ-BF improves the mean SE over the anchored BFNN by 16.23% and 21.55% at 10% and 20% bandwidth, respectively. The gain is larger at 20% bandwidth, indicating a greater benefit from wideband phase correction over the wider of the two evaluated bandwidths.

The BFNN uses wideband CSI, the shared MRT reference, and the common scenario protocol; network configurations are listed in Table 1.

Across the four conditions, CRTQ-BF improves the mean SE by 1.94–7.63% over Sohrabi–Yu. Although OMP uses the less restrictive continuous-phase configuration, CRTQ-BF still achieves an 8.03–25.73% higher mean SE than OMP. Its conditional-tail SE exceeds that of CARAR+QPP by 10.99–16.29%.

#### 4.3.1. SE Versus SNR

**Figure 8:**
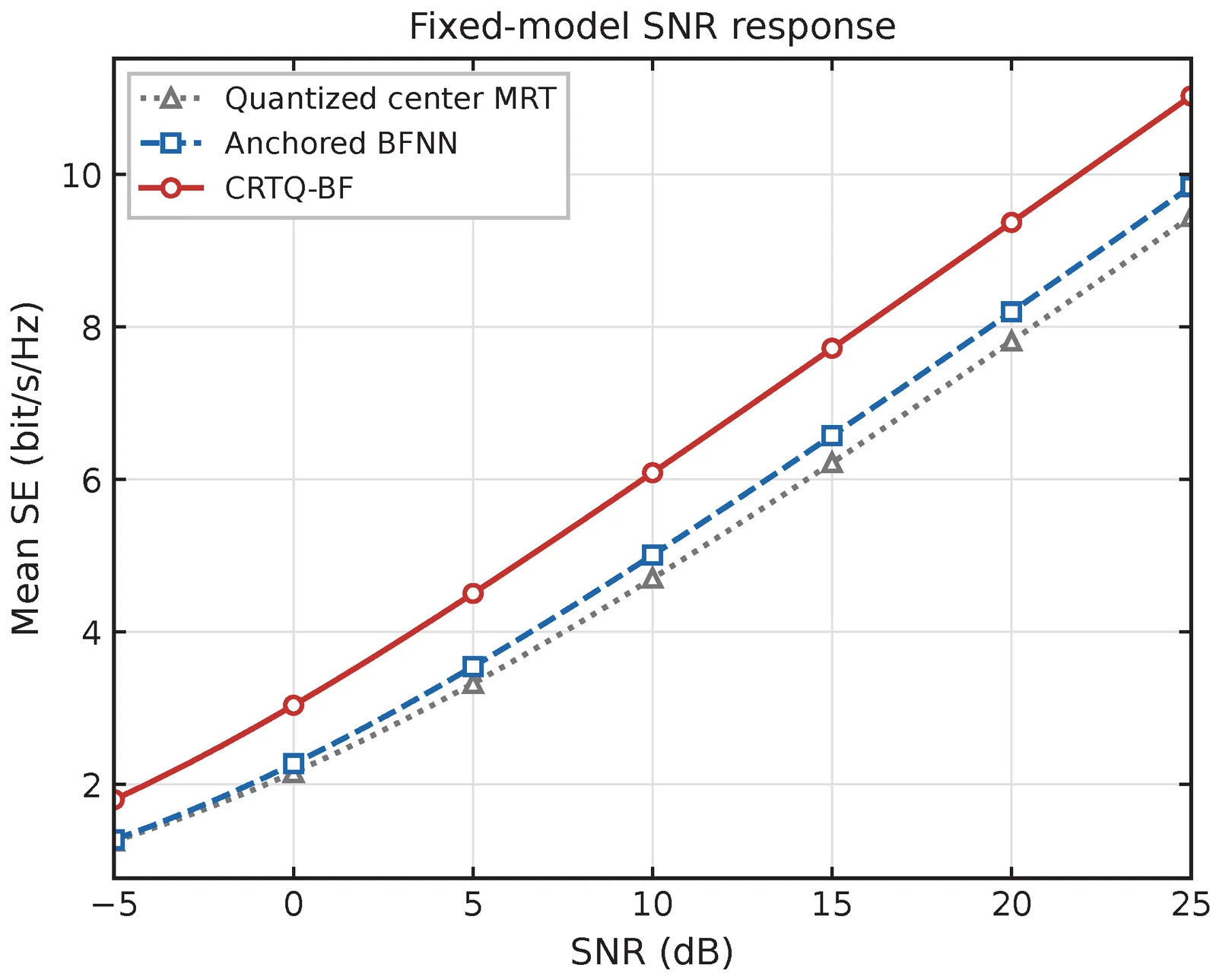
Mean SE versus SNR at 20% bandwidth with 2-bit phases. Models trained at 10 dB are evaluated without retraining.

Figure 8 compares the mean SE of CRTQ-BF, the anchored BFNN, and quantized center-subcarrier MRT at different SNRs. The mean SE of all three methods increases with SNR. At −5, 10, and 25 dB, CRTQ-BF improves the mean SE over the BFNN by 41.20%, 21.55%, and 12.05%, respectively.

#### 4.3.2. Training Convergence and Center-Reference Ablation

**Figure 9:**
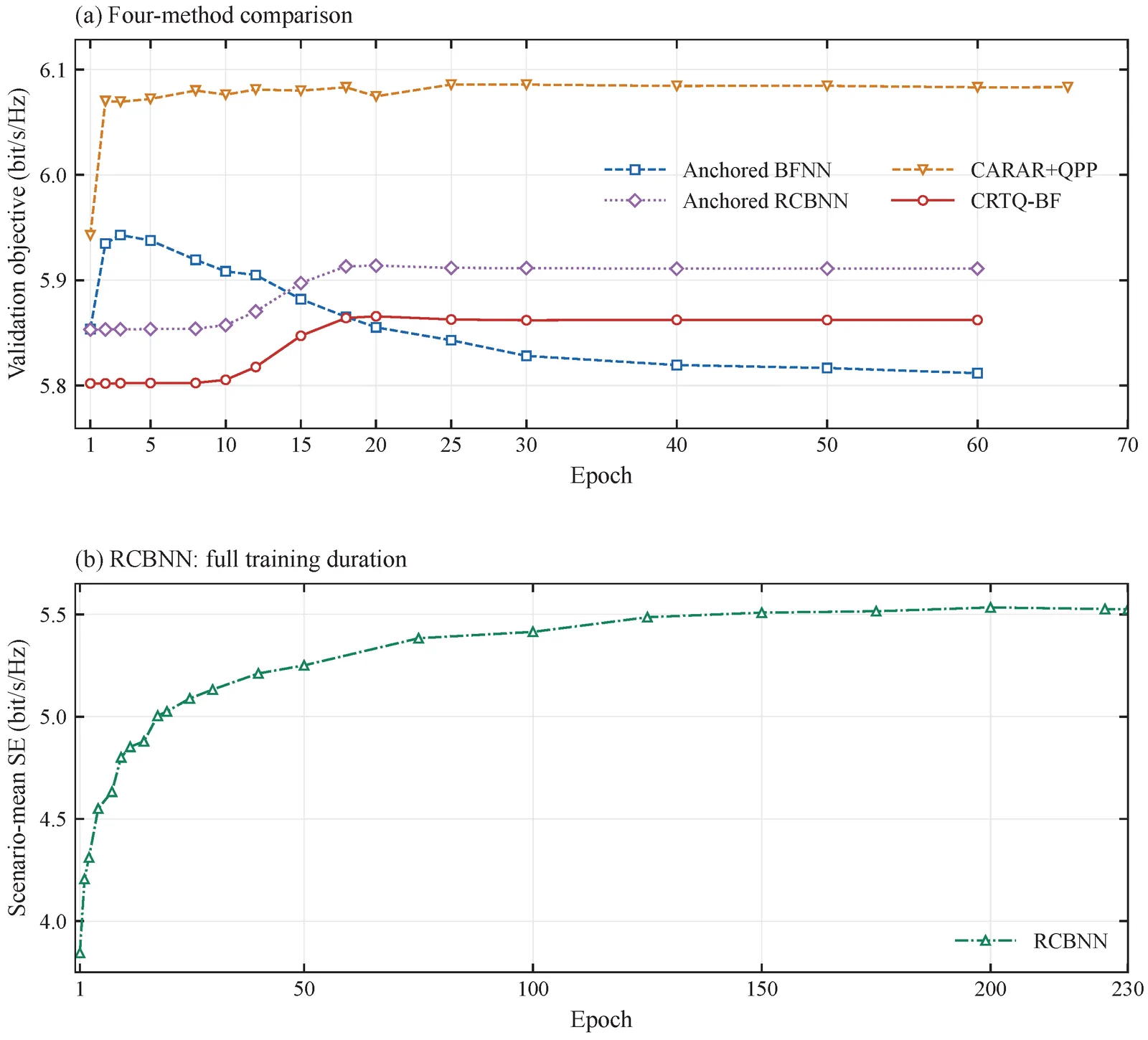
Validation-objective convergence at 10% bandwidth with 2-bit phases: (**a**) anchored BFNN, anchored RCBNN, CARAR+QPP, and CRTQ-BF; (**b**) RCBNN. The BFNN and the RCBNN variants use the scenario-mean SE, CARAR+QPP uses the nominal-CSI mean SE, and CRTQ-BF uses the weighted mean and lower-tail SE objective.

Figure 9 shows validation-objective trajectories under 2-bit control at 10% bandwidth. Figure 9a compares the anchored BFNN, anchored RCBNN, CARAR+QPP, and CRTQ-BF, while Figure 9b shows the complete RCBNN trajectory. The BFNN reaches a validation plateau earlier, whereas RCBNN requires more training epochs. The CARAR+QPP and CRTQ-BF curves stabilize in the later training epochs.

**Figure 10:**
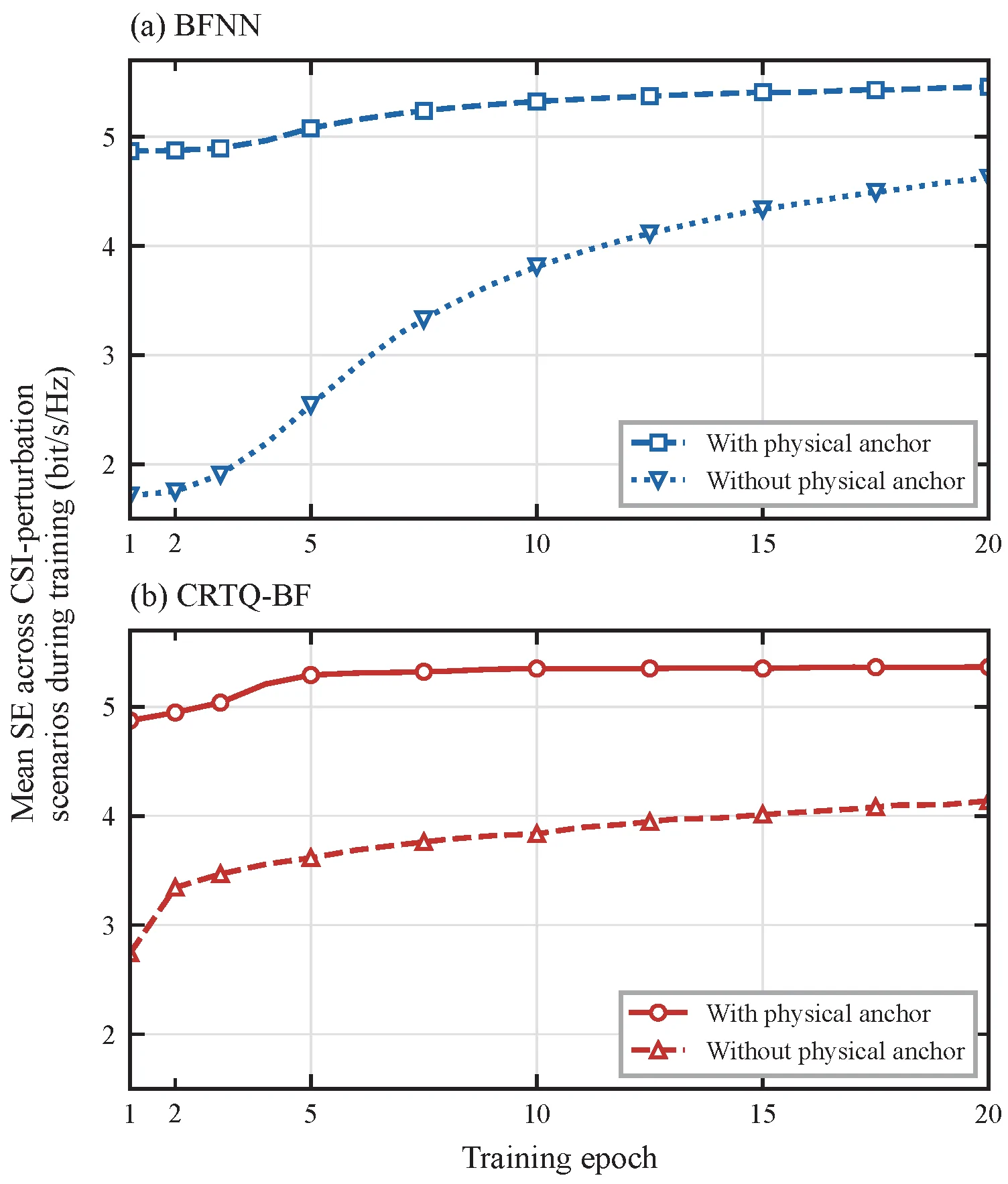
Mean SE across CSI-perturbation scenarios during training: reference ablation (20% BW, 2-bit); (**a**) BFNN; (**b**) CRTQ-BF.

Figure 10 shows the effect of the center-subcarrier reference on the BFNN and CRTQ-BF. For both networks, the reference provides a higher initial mean SE across CSI-perturbation scenarios and maintains this advantage throughout training. In a separate held-out evaluation before phase refinement, adding the reference increases the BFNN’s mean SE from 2.930 to 4.928 bit/s/Hz and CRTQ-BF’s from 3.799 to 5.269 bit/s/Hz. These results show that the center reference provides a useful starting beam and improves test SE under the evaluated training budget.

#### 4.3.3. Test-Channel SE Distribution

**Figure 11:**
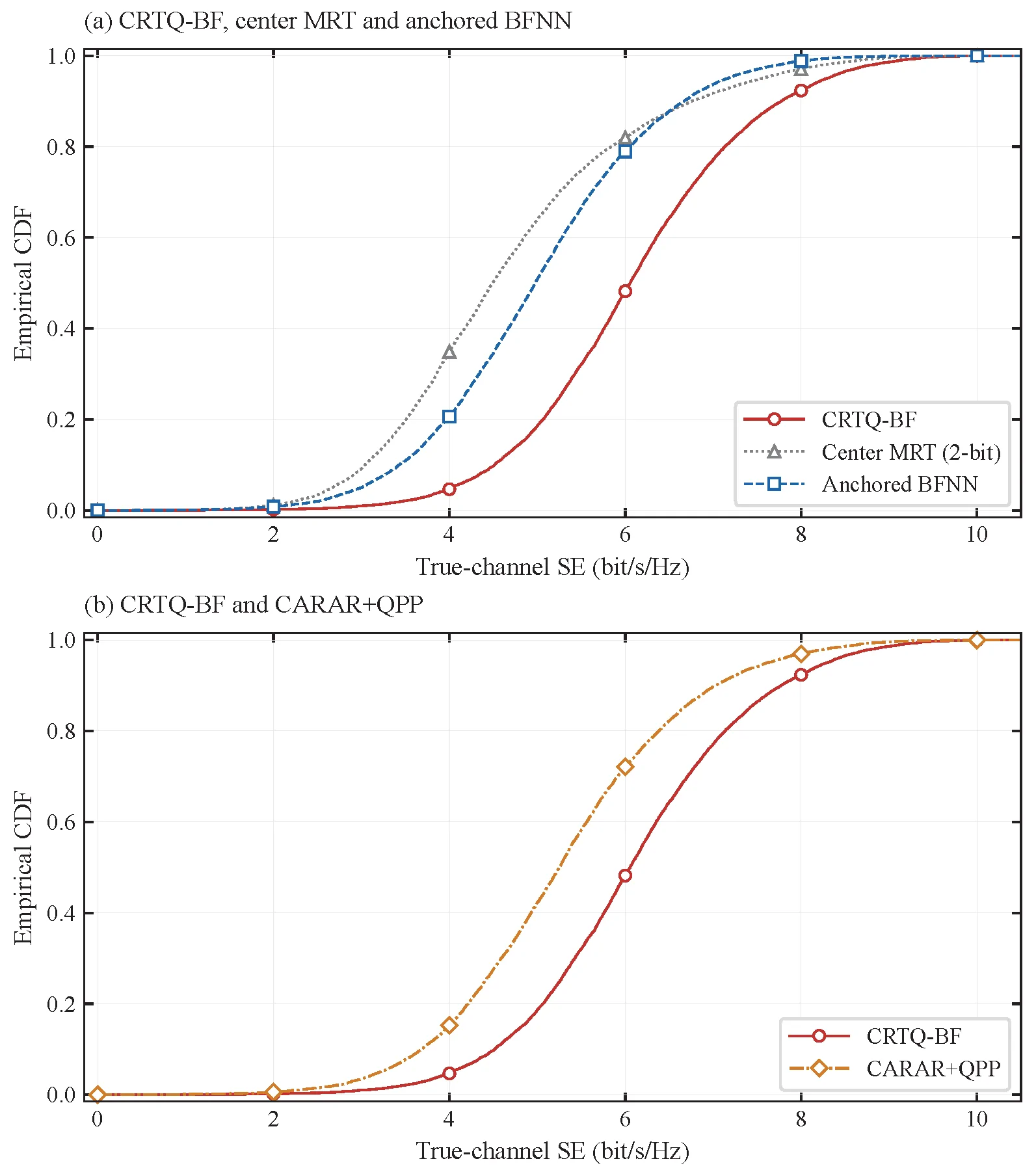
Empirical test-SE CDF at 20% bandwidth, 10 dB, and 2-bit control: CRTQ-BF versus (**a**) MRT and BFNN and (**b**) CARAR+QPP. CRTQ-BF results include TASL discrete phase refinement.

Figure 11 compares test-channel performance using the cumulative distribution function (CDF) of SE. For an SE threshold *r*, the empirical CDF is defined as

(37) $${\hat{F}}_{R}\left(r\right)=\frac{1}{M_{\mathrm{tot}}}\sum_{i=1}^{M_{\mathrm{tot}}}I\left\{R_{i}\leq r\right\},$$

where $R_{i}$ is the SE of the *i*th test channel and $M_{\mathrm{tot}}$ is the total number of pooled test samples. Thus, ${\hat{F}}_{R}\left(r\right)$ is the proportion of test channels with SE at or below *r*; a smaller value at the same threshold indicates fewer low-SE channels.

Figure 11a shows that CRTQ-BF has fewer low-SE test channels than quantized center MRT and the anchored BFNN. At an SE threshold of 4 bit/s/Hz, the cumulative probabilities are 4.67%, 34.88%, and 20.61% for CRTQ-BF, MRT, and the BFNN, respectively. Thus, CRTQ-BF reduces the proportion of channels with SE at or below this threshold. In Figure 11b, the CRTQ-BF distribution is shifted toward higher SE compared with CARAR+QPP.

#### 4.3.4. Beam Gain and Subcarrier Responses

Table 4 reports continuous-phase shared-beam and per-subcarrier reference results. “Shared-beam opt.” uses perfect CSI and Adam to optimize the mean SE of a continuous-phase constant-modulus shared beam, which is initialized from the center MRT beam. Its 2-bit counterpart searches for the phase setting of each antenna within the discrete phase set. The per-subcarrier reference uses a separate beam at each frequency to assess the effect of frequency-dependent beam control.

**Figure 12:**
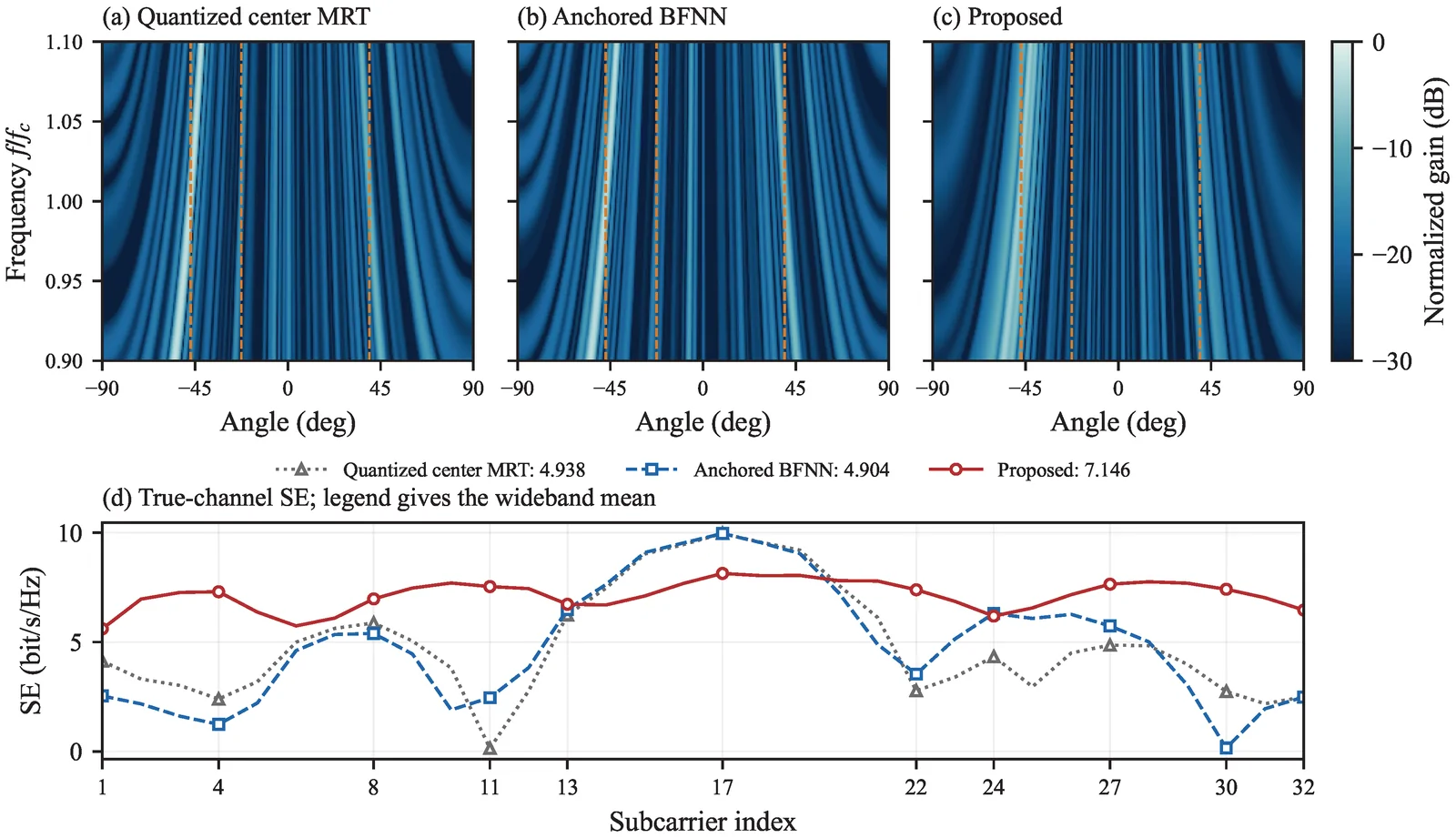
Held-out beam responses under identical 2-bit control: (**a**) quantized center MRT; (**b**) anchored BFNN; (**c**) CRTQ-BF. Orange dashed lines mark path departure angles near $-47^{\circ }$, $-23^{\circ }$, and $40^{\circ }$. The example uses 20% bandwidth, 10 dB SNR, and $\sigma_{e}^{2}=0.1$. (**d**) Corresponding true-channel subcarrier SE values. CRTQ-BF results include TASL discrete phase refinement.

The upper panels of Figure 12 show the normalized transmit array gain across frequency and angle. CRTQ-BF forms broader gain regions near $-47^{\circ }$ and $40^{\circ }$ than quantized center MRT and the anchored BFNN. Relatively high-gain regions remain around both path directions across the subcarriers, extending the angular coverage of the shared beam over the band. This helps maintain gain toward these paths as the beam direction changes with frequency. The example in Figure 12 is the test channel whose anchored-BFNN mean SE is closest to the median.

In Figure 12d, CRTQ-BF achieves 7.537 bit/s/Hz at subcarrier 11 versus 0.143 for MRT and 7.413 at subcarrier 30 versus 0.152 for the BFNN. Its wideband mean is 7.146 bit/s/Hz compared with 4.938 and 4.904 for MRT and the BFNN. Together with the preceding mean-SE and lower-tail-SE results, these findings show that CRTQ-BF improves average SE while enhancing performance in low-SE CSI-perturbation scenarios.

**Figure 13:**
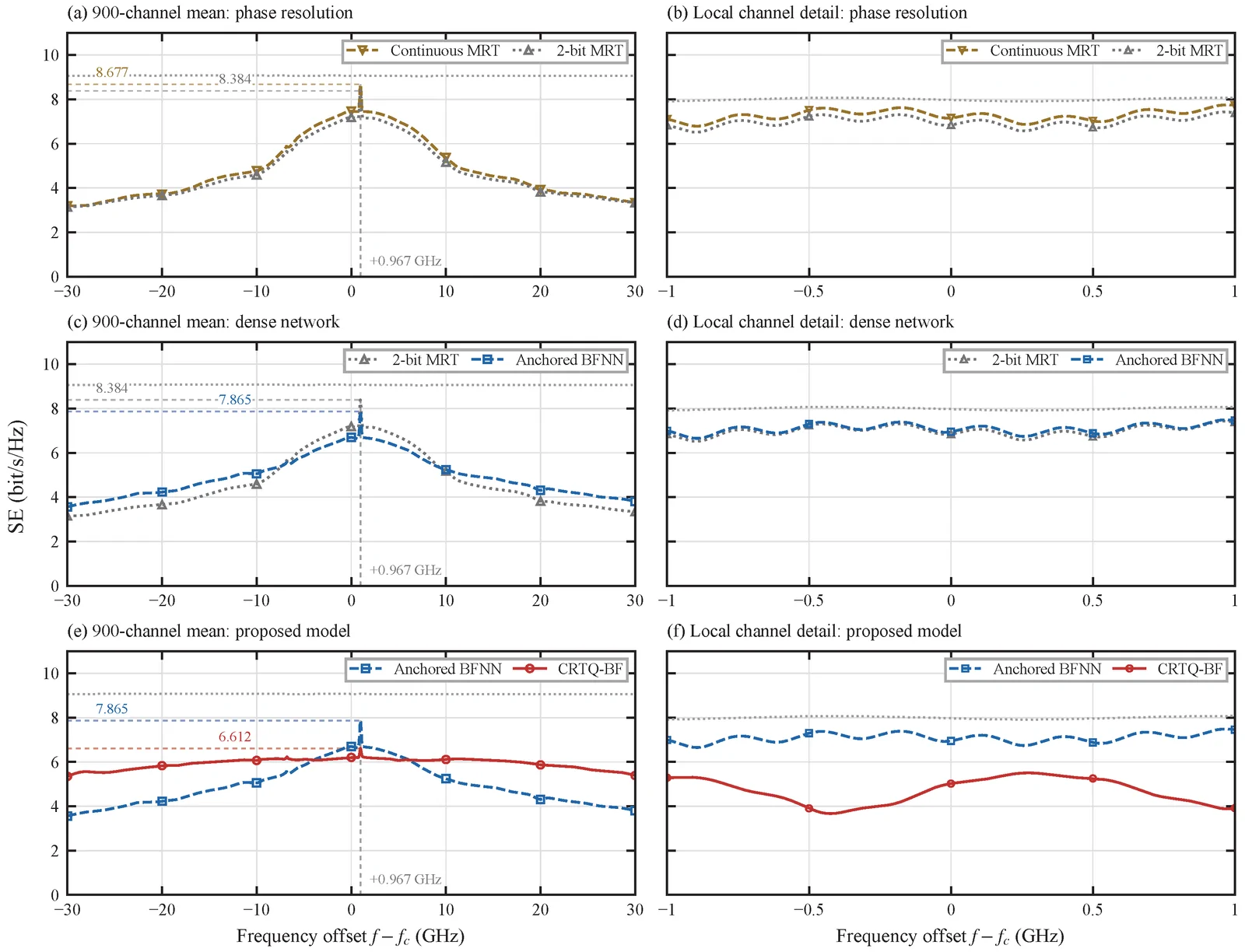
Frequency profiles at 300 GHz and 20% bandwidth: left, 900-channel means; right, single-channel detail. Rows compare phase resolution, BFNN versus MRT, and CRTQ-BF versus BFNN. Gray dotted curves: perfect-CSI digital MRT. CRTQ-BF results include TASL discrete phase refinement.

Figure 13 compares frequency profiles averaged over 900 channels with a single-channel example. The perfect-CSI digital MRT curve provides a reference with per-subcarrier beam control.

### 4.4. Robustness Analysis

We evaluate trained models under changes in CSI error, supplied error variance, and bandwidth.

#### 4.4.1. CSI-Error Sensitivity

**Figure 14:**
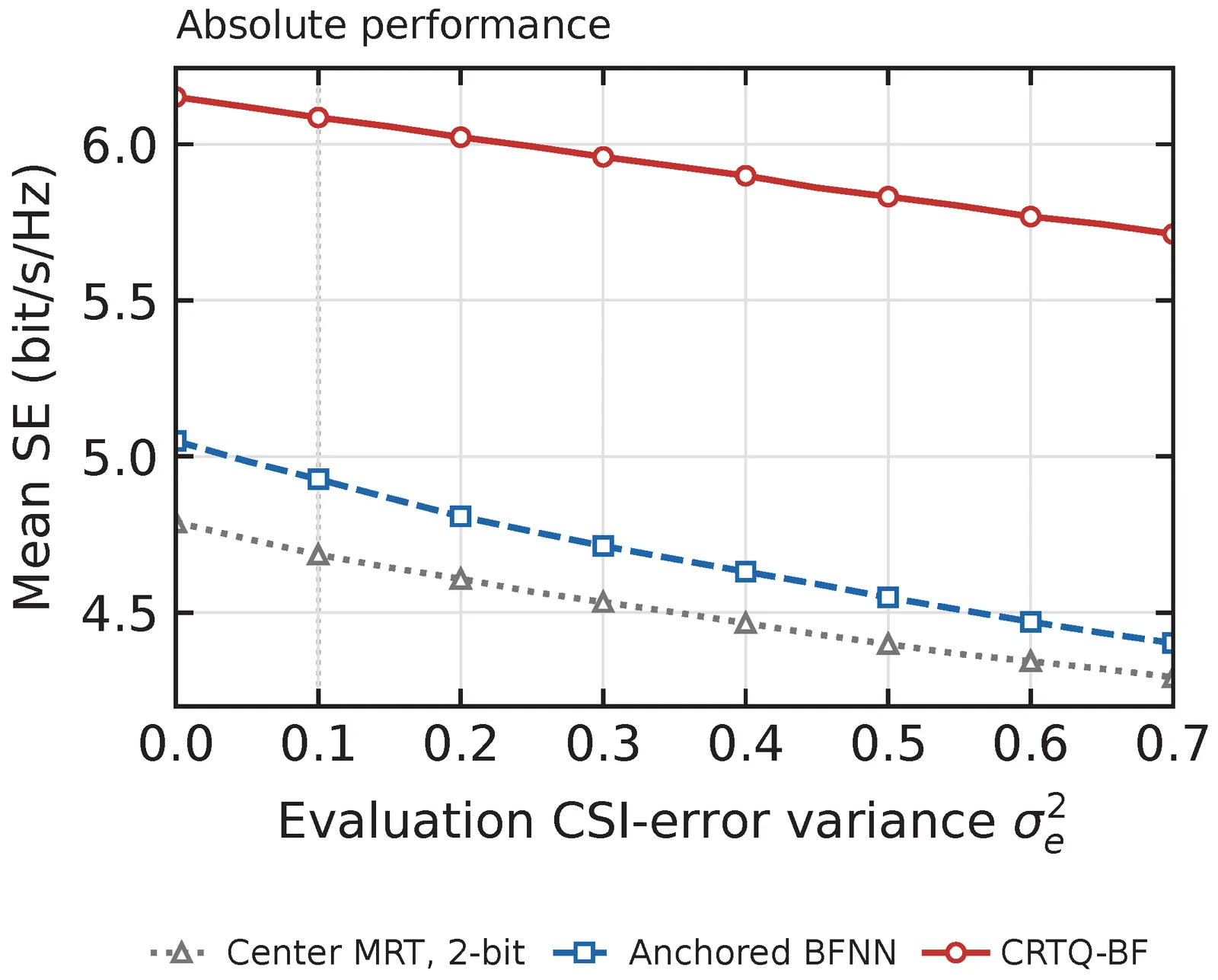
Fixed-model CSI-error sensitivity at 20% bandwidth with 2-bit phases. The dotted line marks the training variance, $\sigma_{e}^{2}=0.1$. The CSI-error variance supplied to the controller equals the variance used to generate the observation errors.

Figure 14 shows the decreasing mean SE with increasing CSI-error variance. CRTQ-BF remains above the BFNN and quantized center MRT over the evaluated range.

**Figure 15:**
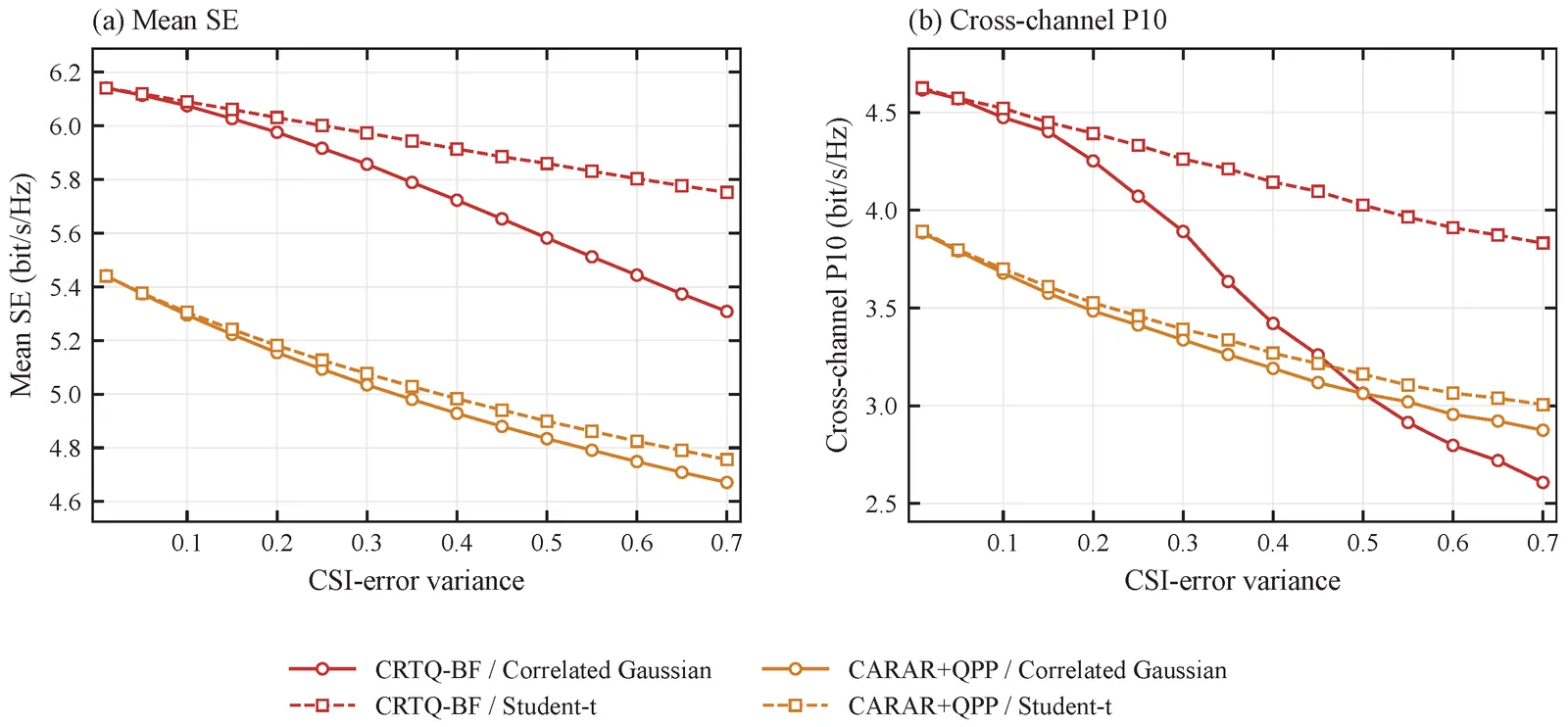
Performance under CSI-error distribution mismatch: (**a**) mean SE; (**b**) cross-channel P10. Both panels compare frequency-correlated Gaussian errors with correlation coefficient 0.8 and variance-matched complex Student-*t* errors with five degrees of freedom. Results use mixed-variance-trained models with 2-bit phases, 20% bandwidth, and 10 dB SNR.

Using models trained with an equally weighted mixture of error variances {0.01,0.1,0.3,0.7}, we further evaluate CRTQ-BF under correlated Gaussian and Student-*t* errors, as shown in Figure 15. CRTQ-BF maintains a mean-SE advantage over CARAR+QPP under the tested conditions; under correlated Gaussian errors with a correlation coefficient of 0.8 and larger error variances, its cross-channel P10 is lower than that of CARAR+QPP.

#### 4.4.2. Supplied Error-Variance Mismatch

**Figure 16:**
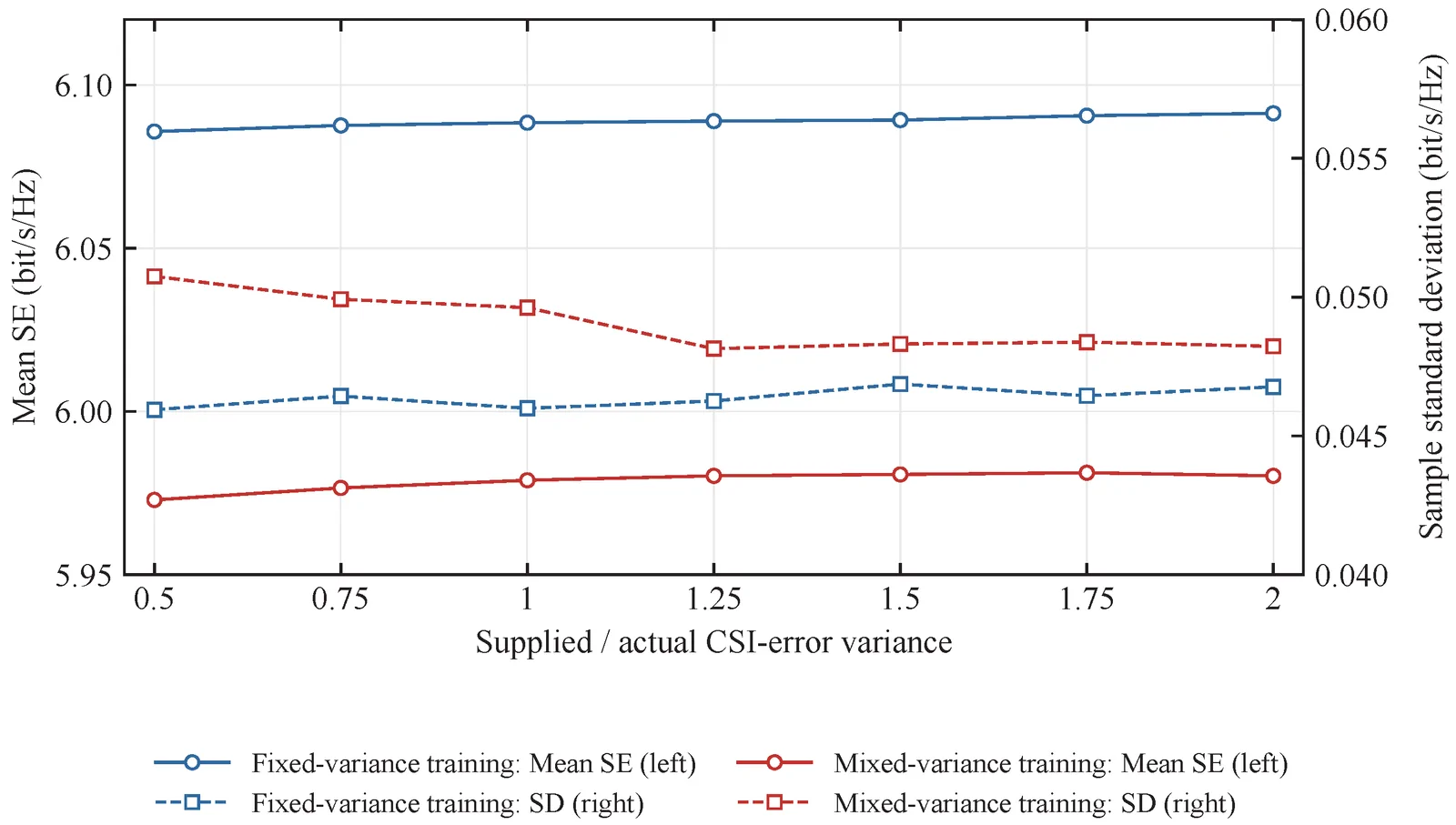
Mean SE (left axis) and sample standard deviation across independent training runs (right axis) of CRTQ-BF under supplied error-variance mismatch, with 2-bit phases, 20% bandwidth, and 10 dB SNR. Training and evaluation use error variance 0.1 in the fixed-variance setting and an equally weighted mixture of {0.01,0.1,0.3,0.7} in the mixed-variance setting. A horizontal-axis ratio of 1 denotes the correctly specified variance input.

To evaluate sensitivity to an inaccurate supplied error variance, we hold the true channels and observed CSI fixed and vary the supplied variance from 0.5 to 2 times its actual value. Figure 16 shows the mean SE and sample standard deviation across independent training runs for both training settings.

Across the tested range, the maximum absolute change in the mean SE relative to the correctly specified input is 0.00602 bit/s/Hz across the fixed-variance and mixed-variance trained models, indicating low sensitivity to variance-input errors within this range.

#### 4.4.3. Joint Bandwidth and CSI-Error Sensitivity

**Figure 17:**
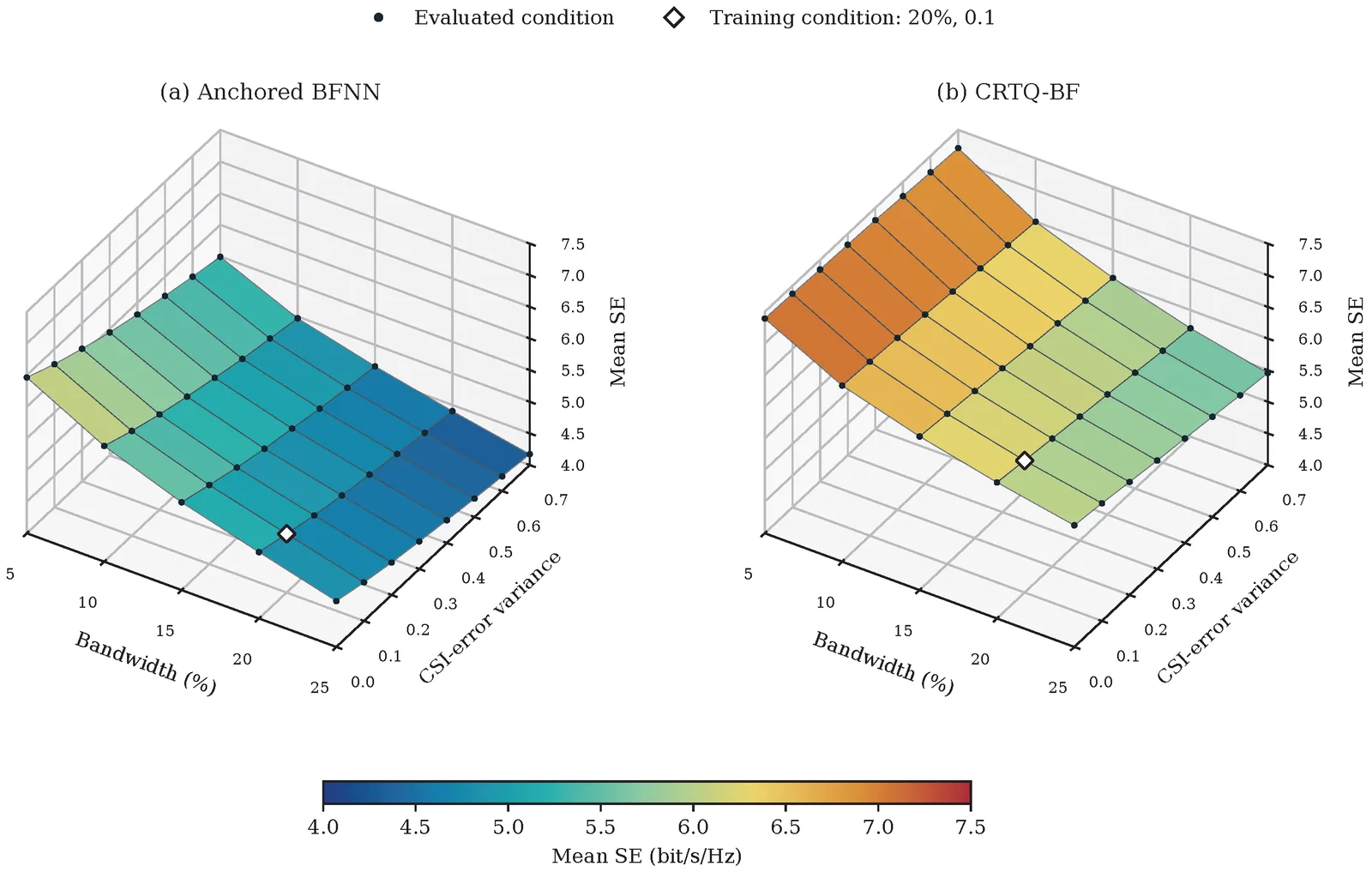
Joint bandwidth–CSI-error response at 10 dB with 2-bit phases: (**a**) anchored BFNN; (**b**) CRTQ-BF. Models are trained at 20% bandwidth and $\sigma_{e}^{2}=0.1$, marked by diamonds, and evaluated without retraining.

In Figure 17, increasing $\sigma_{e}^{2}$ from 0 to 0.7 at 20% bandwidth decreases the mean SE of CRTQ-BF from 6.152 to 5.712 bit/s/Hz; its gain over the BFNN changes from 1.102 to 1.310 bit/s/Hz.

Table 5 summarizes the ranges of the mean SE gains achieved by CRTQ-BF over the BFNN at different fractional bandwidths. CRTQ-BF achieves a higher mean SE across the evaluated combinations of bandwidth and CSI-error variance.

### 4.5. Phase-Resolution Analysis

To assess the effect of phase resolution, we conduct a separate 1–6-bit sweep at fractional bandwidths of 10% and 20% under imperfect CSI. As shown in Table 6, 3-bit control achieves the highest mean SE at both bandwidths, reaching 6.8234 and 6.1169 bit/s/Hz, respectively; 2-bit control provides comparable performance at a lower phase resolution. Therefore, to examine the trade-off between spectral efficiency and phase-shifter resolution requirements, we use 2-bit and 3-bit phase control for the main comparisons.

### 4.6. Complexity Analysis

Table 7 and Table 8 separate the model storage, core arithmetic work, and measured inference time. FP32 denotes 32-bit floating-point representation.

Let *c* denote the convolutional width, $L_{c}$ the number of residual blocks, and *d* the phase-head hidden width with q=⌊d/2⌋ for the second dense-baseline hidden layer. The convolutional and linear multiply–accumulate (MAC) counts per sample are

(38) $$M_{\mathrm{BFNN}}=2KN_{t}d+dq+qN_{t},$$

(39) $$\begin{array}{cc} M_{\mathrm{CRTQ}}= & KN_{t}\left[18c+18L_{c}c^{2}+c\left(c+1\right)\right]\\ & +cN_{t}d+dN_{t}. \end{array}$$

The bracket counts input, residual-block, and gating convolutions. One MAC represents two real operations. Counts exclude biases, normalization, nonlinearities, pooling, and phase mapping.

With $N_{t}=64$, K=32, c=16, $L_{c}=2$, and d=64, CRTQ-BF uses 79,792 parameters versus 274,592 for anchored BFNN, which is a 70.94% reduction. Including BatchNorm buffers, the state-tensor storage decreases from 1,131,144 to 319,848 bytes, which is a reduction of 71.72%. Core MACs increase from 266,240 to 20,090,880 because shared convolutional weights are reused over the frequency–antenna grid. Relative to the same CNN backbone, CRTQ-BF adds 288 parameters and 2.85% more core MACs. Frequency pooling makes its parameter count independent of *K*, although convolutional work still grows with *K*. TASL phase refinement preserves this trainable-parameter count. TASL phase refinement has time complexity $O(N_{t}S_{r}\left(K+\log S_{r}\right))$ and requires $O(S_{r}K+N_{t})$ auxiliary workspace.

Table 8 reports CPU inference time using FP32 on a single-threaded Core i9-14900HX together with the complete single-sample GPU inference time. With 2-bit phase control and a batch size of one, CRTQ-BF takes 1.070 ms per sample on the CPU compared with 0.139 ms for the anchored BFNN. Together with Table 7, these results show that CRTQ-BF reduces parameter storage while requiring more computation and inference time.

### 4.7. Remote State Estimation Performance Analysis

This section evaluates the effect of terahertz beamforming on sensor-data delivery and estimation accuracy through a remote state-estimation simulation. We adopt an intermittent-observation framework [36] and transmit locally filtered state estimates to a remote estimator [37]. The state evolves according to the following scalar random-walk model:

(40) $$x_{t}=x_{t-1}+w_{t},\;z_{t}=x_{t}+v_{t},\;w_{t}∼N\left(0,Q\right),\;v_{t}∼N\left(0,R\right),$$

where $x_{t}$ is the monitored state and $z_{t}$ is the sensor observation. The independent process and measurement noises have variances Q=0.1 and R=1. A local steady-state Kalman filter produces ${\hat{x}}_{t}^{\mathrm{loc}}$ with error variance $\bar{P}=\left(\sqrt{Q^{2}+4QR}-Q\right)/2=0.2702$. At each update, this local estimate is included in the aggregated data frame and transmitted over the terahertz link to the remote estimator.

For the transmission interval after beam configuration, we use an ideal capacity-outage model with coding across subcarriers. The frame reception indicator is

(41) $$\gamma_{t}=1\left\{S_{t}\geq \eta \right\},\;\eta =\frac{L}{WT_{\mathrm{tx}}},$$

where $S_{t}=R\left(v_{t}\right)$ is the true-channel SE in (5) at update *t*, *L* is the aggregate frame payload containing the local state estimate, *W* is the link bandwidth, and $T_{\mathrm{tx}}$ is the allocated transmission duration. The offered spectral load η is measured in bit/s/Hz; $\gamma_{t}=1$ indicates successful reception within this interval. Quantization error in the transmitted local estimate is assumed to be negligible. Received frames are used for the current update, and failed transmissions are not retransmitted. The remote estimator uses the received local estimate when $\gamma_{t}=1$ and otherwise predicts from its previous estimate. Let $P_{t}$ denote the remote estimation error variance at update *t*. With a scalar state-transition coefficient equal to one and negligible quantization of the local estimate, this variance is updated as

(42) $$P_{t}=\left\{\begin{array}{cc} \bar{P}, & \gamma_{t}=1,\\ P_{t-1}+Q, & \gamma_{t}=0. \end{array}\right.$$

Both estimators are initialized to zero with initial error variance $P_{0}=\bar{P}$.

The experiment uses the same settings as above: 2-bit phase control, 20% fractional bandwidth, and 10 dB SNR with identical channel and noise realizations across methods. The link bandwidth is therefore W=60 GHz, and η=4 corresponds to a required transmission rate of $L/T_{\mathrm{tx}}=\eta W=240$ Gbit/s. The offered spectral load is η=1,1.5,…,6, and the CSI-error variances are 0.1,0.3,0.5, and 0.7. Varying η allows us to assess frame delivery success and remote estimation error under different transmission-rate requirements.

**Figure 18:**
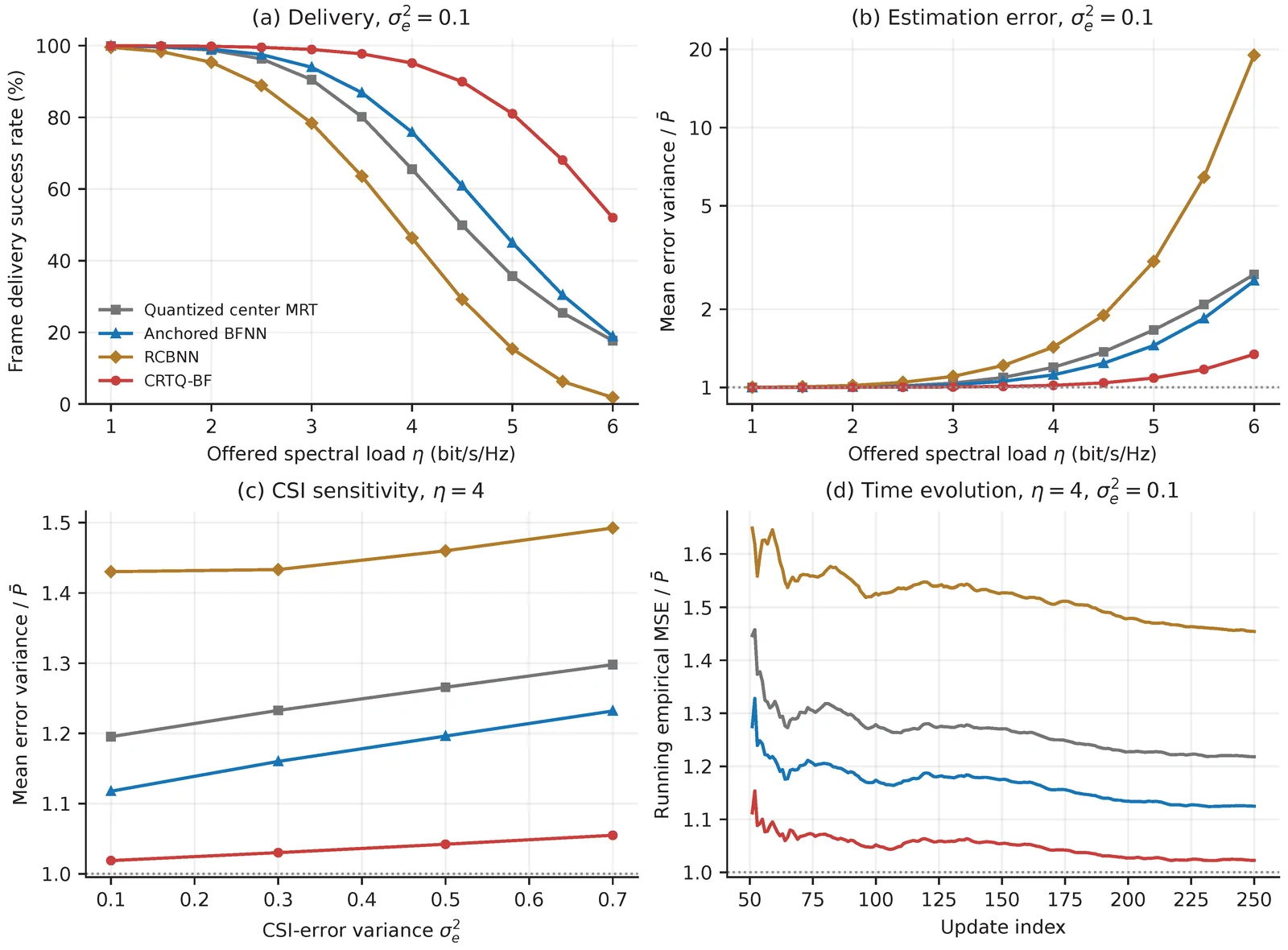
Remote state estimation with quantized beamforming over a terahertz link. (**a**) Frame delivery success rate versus offered spectral load. (**b**) Mean remote error variance versus offered spectral load (logarithmic vertical axis). (**c**) Mean remote error variance versus CSI-error variance at η=4. (**d**) Running empirical MSE at η=4 and $\sigma_{e}^{2}=0.1$. Error metrics in (**b**–**d**) are normalized by the local estimation error variance $\bar{P}$; dotted lines indicate the reference with successful reception at every update.

Figure 18 compares delivery and estimation performance across offered spectral loads and CSI-error levels. At low offered spectral loads, the mean remote error variance approaches $\bar{P}$ for all methods.

As shown in Table 9, at η=4 and $\sigma_{e}^{2}=0.1$, CRTQ-BF increases the frame delivery success rate from 75.87% for the anchored BFNN to 95.16%. The mean remote error variance and empirical MSE of CRTQ-BF are 0.2752 and 0.2762, respectively. Across the experiments with η=4 and CSI-error variances of 0.1,0.3,0.5, and 0.7, CRTQ-BF reduces the empirical MSE of remote estimation by 9.10–15.35% relative to the anchored BFNN.

These results show that CRTQ-BF improves frame delivery success and reduces remote state-estimation error under the tested traffic-load and CSI-error conditions.

## 5. Conclusions

This paper proposes CRTQ-BF for wideband terahertz sensor-data transmission from a multiantenna transmitter to a single-antenna receiver. The design addresses imperfect CSI and finite-resolution phase shifters in a single-RF-chain MISO link. The algorithm integrates CARAR, TASL, and QPP to learn one analog phase vector shared across all subcarriers. CARAR combines the center-subcarrier phase reference with reliability-conditioned channel features to learn a wideband phase correction. TASL gives greater training weight to low-SE CSI-perturbation scenarios while retaining the mean-SE objective and refines the quantized beam within the finite phase alphabet at deployment. QPP enforces discrete phase settings in the forward pass and passes a surrogate gradient backward, enabling the joint training of the phase-correction network under finite-resolution constraints.

Simulation results show that CRTQ-BF improves the mean SE over the anchored BFNN under the evaluated wideband conditions while requiring fewer trainable parameters and less model storage. The algorithm also maintains SE gains under increased CSI error and exhibits low sensitivity to supplied error-variance mismatch. The additional remote-estimation simulation shows that improved frame delivery translates into lower estimation MSE, supporting accurate remote monitoring over the modeled sensor-data forwarding link. At an offered spectral load of 4 bit/s/Hz, the MSE reduction over the anchored BFNN is 9.10–15.35% across the evaluated CSI-error variances.

Future work will use device measurements to model the amplitude response and phase errors of practical phase shifters at each discrete setting and include these responses in network training. We will also evaluate measured gateway channels and sensor traffic to assess deployment conditions and application-level performance, and investigate network compression and inference acceleration to reduce beam computation latency.

## Figures and Tables

**Table 1:** Neural architectures implemented for the controlled comparisons. RCBNN denotes the residual convolutional neural network (CNN) beamforming baseline implemented here for the wideband setting. The BFNN baseline is adapted from [21]; the convolutional backbone uses residual learning [32]. All methods use exact forward phase projection and an STE; CARAR and CRTQ-BF use the reliability gate. BatchNorm denotes batch normalization, and MLP denotes multilayer perceptron.

Method	Architecture
Anchored BFNN	Flattened $2\times K\times N_{t}$ input; BatchNorm; fully connected layers 4096→64→32→64; residual phase head.
RCBNN	3×3 convolution 2→16; BatchNorm and ReLU; two residual blocks with two 3×3 convolutions 16→16 per block; frequency mean pooling; MLP head 1024→64→64; direct phase prediction without a center reference; mean-SE training.
Anchored RCBNN	The same residual CNN with a center-subcarrier phase reference and a zero-initialized residual phase head; mean-SE training. Internal control.
CARAR	Center reference and zero-initialized residual head; same residual backbone; reliability matrix concatenated with features; 1×1 gate convolution 17→16; frequency mean pooling; MLP head 1024→64→64.
CRTQ-BF	The CARAR architecture with the tail-aware scenario learning objective. Deployment additionally applies TASL phase refinement; this adds no trainable parameters.

**Table 2:** Simulation and training parameters.

Parameter	Setting
Transmit antennas $N_{t}$	64
Receive antennas	1
RF chains	1
Subcarriers *K*	32
Channel model	Saleh–Valenzuela
Number of paths *L*	3
Fractional bandwidth (BW)	10%, 20%
SNR	10 dB
CSI error variance	0.1
Training samples per run	8192
Validation samples	1024
Test samples per run	2048
Repeated runs	5
CSI-uncertainty scenarios	32
Batch size	64
Learning rate (initial)	$5\times 10^{-4}$
Tail fraction α	0.1
Mean/tail weight λ	0.5
Scenario protocol	Estimated CSI plus sampled perturbations only
Phase resolution	2-bit, 3-bit
Deployment scenarios $S_{r}$	128
Deployment design SNR $\rho_{d}$	10 (10 dB)
Evaluation perturbation scenarios	512

**Table 3:** Comparison of the mean SE, P10, and conditional-tail SE across phase resolutions and fractional bandwidths.

Bits	BW	Method	Mean SE	P10	Conditional Tail
2	10%	Anchored BFNN	5.804±0.051	4.133±0.083	5.749±0.052
		Anchored RCBNN	5.864±0.112	4.226±0.104	5.803±0.112
		CARAR+QPP	5.973±0.047	4.330±0.042	5.912±0.047
		CRTQ-BF	6.746±0.046	5.227±0.058	6.730±0.046
		Sohrabi–Yu [13]	6.474±0.050	4.777±0.025	6.455±0.050
		OMP [35] ^†^	6.245±0.049	4.441±0.036	6.215±0.050
2	20%	Anchored BFNN	5.009±0.057	3.431±0.024	4.962±0.055
		Anchored RCBNN	5.297±0.044	3.684±0.026	5.238±0.045
		CARAR+QPP	5.300±0.045	3.692±0.037	5.240±0.045
		CRTQ-BF	6.088±0.046	4.511±0.033	6.093±0.046
		Sohrabi–Yu [13]	5.657±0.051	3.885±0.026	5.675±0.051
		OMP [35] ^†^	4.877±0.043	3.041±0.027	4.914±0.043
3	10%	Anchored BFNN	6.006±0.046	4.338±0.044	5.950±0.047
		Anchored RCBNN	6.180±0.048	4.526±0.033	6.120±0.048
		CARAR+QPP	6.178±0.049	4.519±0.036	6.118±0.049
		CRTQ-BF	6.812±0.043	5.252±0.045	6.791±0.043
		Sohrabi–Yu [13]	6.682±0.048	4.944±0.030	6.664±0.048
		OMP [35] ^†^	6.245±0.049	4.441±0.036	6.215±0.050
3	20%	Anchored BFNN	5.178±0.056	3.580±0.048	5.130±0.055
		Anchored RCBNN	5.492±0.046	3.859±0.046	5.431±0.046
		CARAR+QPP	5.491±0.046	3.865±0.052	5.430±0.047
		CRTQ-BF	6.132±0.047	4.529±0.023	6.127±0.046
		Sohrabi–Yu [13]	5.847±0.044	4.093±0.018	5.866±0.044
		OMP [35] ^†^	4.877±0.043	3.041±0.027	4.914±0.043

SNR: 10 dB; CSI-error variance: 0.1. Values are presented as mean ± standard deviation in bit/s/Hz. † OMP retains the continuous-phase output of the original method and is not subject to the 2-bit or 3-bit phase quantization indicated in the table; the other methods use the indicated phase resolution.

**Table 4:** Separate physical-reference experiment. The per-subcarrier phase-only oracle uses perfect CSI and an independent equal-amplitude beam at each frequency. Values are presented as mean ± standard deviation.

BW	Reference	Mean	P10
10%	Center MRT (2-bit)	5.941 ± 0.025	4.319 ± 0.153
10%	Center MRT	6.168 ± 0.029	4.471 ± 0.173
10%	Phase-only oracle	8.872 ± 0.026	7.766 ± 0.039
10%	Shared-beam opt.	7.263 ± 0.015	5.809 ± 0.072
10%	Shared-beam opt. (2-bit)	6.057 ± 0.031	4.420 ± 0.170
20%	Center MRT (2-bit)	4.874 ± 0.024	3.187 ± 0.048
20%	Center MRT	5.022 ± 0.024	3.209 ± 0.140
20%	Phase-only oracle	8.873 ± 0.026	7.767 ± 0.036
20%	Shared-beam opt.	6.633 ± 0.015	5.210 ± 0.168
20%	Shared-beam opt. (2-bit)	5.051 ± 0.028	3.410 ± 0.065

**Table 5:** Range of mean SE differences over the eight CSI-error variances in the fixed-model joint sweep with TASL deployment refinement (bit/s/Hz).

BW (%)	CRTQ-BF − BFNN
5	[0.932,1.718]
10	[0.954,1.528]
15	[1.041,1.397]
20	[1.102,1.310]
25	[1.197,1.293]

**Table 6:** Mean SE of CRTQ-BF at different phase resolutions. Values are presented as mean ± sample standard deviation, in bit/s/Hz.

Phase Resolution	10% Bandwidth	20% Bandwidth
1 bit	5.9714±0.0764	5.3446±0.0527
2 bits	6.7720±0.1006	6.0856±0.0798
3 bits	6.8234±0.0983	6.1169±0.0808
4 bits	6.6670±0.1060	5.9579±0.0945
5 bits	6.4968±0.1136	5.7836±0.1031
6 bits	6.3786±0.1164	5.6636±0.1093

**Table 7:** Model size and core multiply–accumulate counts.

Method	Parameters	State (KiB)	MACs ($10^{6}$)
Anchored BFNN	274,592	1104.63	0.266
Anchored CNN control	79,504	311.23	19.534
CRTQ-BF	79,792	312.35	20.091

$N_{t}=64$, K=32. State includes FP32 parameters and BatchNorm buffers; MACs count only convolutional and linear layers.

**Table 8:** Comparison of CPU network inference and complete GPU inference times (ms/sample).

CPU: Network Inference to the Initial QPP Beam				
Bits	Method	Batch Size 1	Batch Size 16	Batch Size 64
2	Anchored BFNN	0.1393	0.0170	0.0099
2	Anchored CNN control	0.9433	0.5366	0.6087
2	CRTQ-BF	1.0700	0.6362	0.7386
3	Anchored BFNN	0.1397	0.0170	0.0098
3	Anchored CNN control	0.9312	0.5395	0.6042
3	CRTQ-BF	1.0622	0.6418	0.7179
**GPU: Complete Inference, Batch Size 1**				
**Bits**	**Method**	**Time (Mean ± Sample Standard Deviation)**		
2	Anchored BFNN	0.7881±0.0324		
2	CRTQ-BF	8.0517±0.2104		

Core i9-14900HX, one thread, FP32. Median of three checkpoint medians; seven warmed blocks per checkpoint. For batch sizes greater than one, the reported time is the mean time per sample, which is calculated by dividing the total batch processing time by the batch size. GPU: RTX 4070 Laptop. Timing covers data transfers and the complete beam-computation pipeline.

**Table 9:** Remote state-estimation results at η=4, $\sigma_{e}^{2}=0.1$, Q=0.1, and R=1. Results are averaged over three training seeds with 64 independent trajectories per seed; the first 50 of 250 updates are discarded.

Method	Frame Delivery Success Rate (%)	Mean Error Variance	Empirical MSE
Quantized center MRT	65.54	0.3229	0.3291
Anchored BFNN	75.87	0.3019	0.3039
RCBNN	46.35	0.3863	0.3929
CRTQ-BF	95.16	0.2752	0.2762

## Data Availability

The data presented in this study were generated synthetically according to the channel, CSI-error, and training settings described in the paper. No external dataset was used. The supporting data, experimental code, and reproduction instructions are openly available in the Supplementary Material (Dataset S1).

## Supplementary Materials

The following supporting information can be downloaded at: https://www.mdpi.com/article/10.3390/s26217081/s1, Dataset S1: Supporting data, experimental code, and reproduction instructions.
