# Supplementary material for: Tail-Aware Unsupervised Learning for Wideband Terahertz Quantized Beamforming in Sensor Data Transmission
Source: Sensors (Basel). 2026 Nov 9;26(21):7081. doi: 10.3390/s26217081 (PMC13766379; doi:10.3390/s26217081)
Supplement: Supplementary file 1 [file sensors-26-07081-s001.zip › Dataset_S1_Guide.pdf]

Dataset S1
Experimental settings and data guide

Manuscript ID: sensors-4635078 | November 1, 2026

Tail-Aware Unsupervised Learning for Wideband Terahertz Quantized Beamforming in Sensor Data Transmission

This supplement records experimental settings, numerical data, file definitions, and instructions for running the supplied experiment code. Figure and table numbers refer to the article; tables in this guide use the prefix S.

1. Package contents

The code directory contains programs for training, beam computation, performance evaluation, timing, and statistical analysis. The data directory contains numerical results and model checkpoints. The file index, data/file_index.csv, lists the files and their fields.

Table S1. Data directory index.

Directory	Experiment / manuscript location
data/main_comparisons	Table 4; Figures 9 and 11; per-seed and per-input metrics, training curves, and actual run settings.
data/fig08_snr	Figure 8: fixed-model SNR sweep, raw received powers and per-seed spectral efficiency.
data/fig10_anchor	Figure 10: center-reference ablation; seeds 101-103, 20 training epochs.
data/fig12_beam	Figure 12: beam responses and subcarrier SE for one test channel; seed 101, sample index 129.
data/fig13_frequency_900	Figure 13: frequency responses averaged over 900 channels (seeds 101-103), a single-channel example, and coordinates of the plotted curves.
data/fig14_csi	Figure 14: CSI-error variance sweep with fixed trained models and deployment refinement.
data/fig15_distribution_mismatch	Figure 15: error-variance sweeps under independent Gaussian, frequency-correlated Gaussian, and Student-t errors; per-input and per-seed results.
data/fig16_variance_input	Figure 16: ratios of supplied to actual variance from 0.5 to 2. Results at ratio 1 are stored in main_comparisons/arrays/fixed_mixed.
data/fig17_joint	Figure 17 and Table 6: joint bandwidth and CSI-error variance sweep. Refined CRTQ-BF results are stored in historical_refined/figure_data.npz under fields beginning with fig15_.
data/fig18_remote	Figure 18 and Table 10: frame reception and remote estimation metrics. Fields beginning with new_ contain CRTQ-BF results after refinement; table10_per_seed.csv and table10_summary.csv provide the table values.
data/historical_baselines	Baseline training curves and CSI-error results for Figures 10 and 14; seeds 101-103.
data/historical_refined	CRTQ-BF results after deployment refinement for Figures 12-14, 17, and 18. Field prefixes fig15 and fig16 identify Figures 17 and 18, respectively.
data/table05_physical	Table 5: numerical references for shared-beam optimization and per-subcarrier phase-only beamforming; results for individual seeds.
data/table07_resolution	Table 7: models trained separately with phase resolutions from 1 to 6 bits; results for each seed, including beam weights and SE values.
data/table08_complexity	Table 8: parameter counts, model storage, and multiply-accumulate (MAC) counts for convolutional and linear layers.
data/table09_timing	Table 9: CPU network inference and complete GPU inference with batch size 1. GPU results use the fixed-variance rows bfnn_host and crtq_S128_host.

Table S1. Data directory index.

Directory	Experiment / manuscript location
data/supplementary/carar	CARAR replacement with matched epoch or time budgets; training time and performance for each seed.
data/supplementary/paired_statistics	Paired statistical analysis: seed-level measurements, paired differences, confidence intervals, and Holm-adjusted paired t tests.
data/supplementary/sensitivity	Sensitivity to training parameters alpha and lambda and deployment weight beta_r; results for individual seeds and inputs.
data/supplementary/scenario_count	Deployment scenario counts Sr = 32, 64, 128, 256: SE and timing data. SE evaluation uses 16 inputs for each of two trained models.
data/supplementary/los	Synthetic channels with one direct path and two reflected paths; two reflection attenuation levels and two CSI-error variances. Geometry and beams are provided for all 20 combinations of seed, attenuation, and variance.
data/checkpoints	115 model checkpoints selected by validation performance: 95 for the main comparisons and 20 for mixed-variance training. Each checkpoint has an accompanying configuration file.
data/fig02_beam_squint	Figure 2: analytical angular response of the 64-element array.

2. Main method comparisons

Table S2. Channel and training settings.

Item	Setting
Carrier frequency; antennas; design frequency points	300 GHz; 64; 32
Multipath parameters	3 paths; angles uniform on [-pi/2, pi/2]; delays uniform on [0,5 ns]; circular complex Gaussian gains with variance 1/3 per path.
Phase resolution and fractional bandwidth	2 bits and 3 bits; 10% and 20%; four combinations
Design SNR and default evaluation SNR	10 dB
Inputs per run	8192 training; 1024 validation; 2048 evaluation
Training seeds	1101, 1102, 1103, 1104, 1105
Fixed training error variance	0.1
Mixed-variance training (2 bits, 20%)	Equal mixture of 0.01, 0.1, 0.3, 0.7
Optimizer; initial learning rate	Adam; 0.0005
Minibatch size; gradient norm limit	64; 5
Training scenarios; tail fraction; mean weight	32; 0.1; 0.5
Model selection	Save the model when the validation objective exceeds the best saved value by more than 0.0001.
Learning-rate schedule	ReduceLROnPlateau: mode=max, factor=0.5, patience=8, absolute threshold=0.0001, minimum rate=0.000001.
Training termination	After at least 60 epochs, stop if 30 consecutive validation checks do not improve the best saved objective by more than 0.0001. The maximum epoch count is specified in each configuration.
Deployment refinement	128 fixed Gaussian scenarios; lower tail consists of the 13 lowest wideband SE values; lower-tail weight 0.5; one pass over the antennas in index order.

Table S2. Channel and training settings.

Item	Setting
Phase update	Compare the current phase, the previous discrete phase, and the next discrete phase, in that order, with wraparound. Keep the first candidate in a tie; update only when the score increases by more than 0.000001.
Conditional evaluation	512 independent Gaussian scenarios per input; average the 52 lowest wideband SE values to obtain the lower-tail mean.

CARAR+QPP uses mean SE under estimated CSI for training and validation. BFNN, RCBNN and anchored RCBNN use the scenario-mean objective. CRTQ-BF uses the weighted scenario mean and lower-tail mean. In the CARAR replacement experiment, both networks use the same weighted mean-and-lower-tail objective.

The center-anchored residual phase head is initialized to zero. The reliability gate is initialized as an identity mapping. Matching backbone parameters use the same initialization within each paired run. Training scenarios are resampled each epoch, while validation scenarios use a fixed random stream.

The Sohrabi-Yu baseline uses a single-RF-chain, single-stream coordinate update, an equal-phase initialization, at most 300 sweeps and the specified discrete phase set. OMP uses the estimated center-subcarrier channel and a 256-direction array-response dictionary, with continuous steering phases. Sources: Sohrabi and Yu (2017), DOI:10.1109/JSAC.2017.2698958; El Ayach et al. (2014), DOI:10.1109/TWC.2014.011714.130846.

3. Random streams and numerical definitions

Table S3. Random-stream rules for the main comparisons.

Random quantity	Seed or rule
Training / validation / evaluation channels	s; s + 1,000,000; s + 2,000,000
Model initialization	s + 5000
Minibatch order at epoch e	s + 10,000e
Training perturbations at epoch e	s + 10,000e + 500,000
Observation-error generator	NumPy generator: channel-data seed + 200,000
Mixed-variance permutation	Channel-data seed + 100,000
Evaluation on independently generated channels	73,001,101 to 73,001,105

Here s denotes the training seed. Deployment selection and conditional evaluation use separate random streams. Scenario generation depends on the seed and batch dimensions; the main method comparisons use batches of eight inputs for evaluation. The corresponding seed rules are specified in the experiment configurations and code.

Table S4. Metric and field definitions.

Field	Definition
mean_se	For each true channel, average SE over frequencies, then average across inputs; unit: bit/s/Hz.
p10_se	Linearly interpolated 10th percentile of the true-channel SE values within one run; bit/s/Hz.
conditional_mean	Mean of the frequency-averaged rates across the independent perturbation scenarios and inputs.
conditional_tail	For each input, average the lowest ceil(0.1S) wideband SE values among S evaluation scenarios, then average across inputs.
outage_below_4	Fraction of inputs with true-channel SE below 4 bit/s/Hz.
sample_sd	Sample standard deviation across n independent runs, calculated with denominator n-1.

Table S4. Metric and field definitions.

Field	Definition
true_rates / beams	SE for each true channel / complex beam weights. The methods field and array dimensions identify the methods and samples.

Methods are paired by training seed. Paired t-tests use the differences between paired runs and report two-sided p-values. The 95% confidence intervals use the Student t distribution and are calculated separately for each comparison. P-values are Holm-adjusted across the 120 comparisons for each metric. The dataset contains five metrics and 600 comparisons in total.

In the main comparison files, bfnn denotes anchored BFNN; rcbnn denotes RCBNN without the physical anchor; anchored_rcbnn denotes anchored RCBNN; no_tasl denotes CARAR+QPP; and crtq denotes the initial QPP output of the network trained with the mean-and-lower-tail objective. The suffix _mix denotes deployment refinement with the joint mean/tail score; _mean denotes mean-only refinement. Model identifiers are retained in the data so that initial and refined results remain distinct.

The result files contain SE values for individual inputs and summary metrics over the perturbation scenarios. The 512 individual scenario rates per input are computed by the evaluation code rather than stored in the main comparison files. Array names and dimensions are listed in data/file_index.csv.

4. Additional experiment settings

Table S5. Settings by experiment.

Experiment	Settings
Error-distribution mismatch	2 bits, 20% bandwidth; models trained with mixed error variances; independent Gaussian, correlated Gaussian (frequency correlation 0.8) and Student-t (5 degrees of freedom, matched marginal variance); 15 variance values.
Supplied-variance mismatch	Ratios of supplied to actual variance: 0.5, 0.75, 1, 1.25, 1.5, 1.75, 2. Hold true channels, observations and actual variance fixed. Models trained with fixed or mixed error variances; five training seeds.
Training-parameter sensitivity	2 bits, 20%; mixed variance; seeds 1102-1105. Vary alpha in {0.05,0.1,0.2} at lambda=0.5, and lambda in {0.25,0.5,0.75,1} at alpha=0.1. Six distinct settings; evaluate initial QPP outputs.
Deployment-weight sensitivity	beta_r = 0, 0.5, 1; five training seeds; fixed and mixed regimes; 128 inputs per model/regime; 128 selection and 512 independent evaluation scenarios.
Deployment scenario count	Sr = 32,64,128,256; models trained with seeds 1101 and 1102; 16 inputs per model; two scenario sets per model. Each set contains 256 scenarios; smaller counts use the first Sr scenarios of that set. Evaluation uses 512 independent scenarios.
CARAR replacement and budgets	Replace CARAR with unanchored RCBNN; retain QPP, mean/tail training and deployment refinement. Five paired seeds; 2 bits, 20%; evaluate variances 0.01,0.1,0.3,0.7. Match RCBNN epochs or elapsed training time to its paired CARAR run.
Dominant direct-path channel	One direct and two reflected paths; reflection attenuation 5 or 10 dB; error variance 0.1 or 0.7; fixed and mixed training; five seeds; 256 inputs per condition; beam design at 32 frequency points and SE evaluation at 1024 frequency points.
Phase-resolution sweep (Table 7)	1-6 bits; 10% and 20% bandwidth; seeds 101-103; a separate model trained for 20 epochs on 1000 inputs per configuration, with 32 fixed scenarios per input; 300 test channels per seed; 128-scenario deployment refinement.
Remote estimation (Figure 18, Table 10)	Seeds 101-103; 64 trajectories per seed; 250 updates, discard first 50; Q=0.1,R=1; error variances 0.1,0.3,0.5,0.7; offered loads 1,1.5,...,6; 2 bits, 20%, 10 dB.

Training time in the CARAR budget comparison includes training, validation, and checkpoint saving. For time-limited runs, the model is selected by validation performance from epochs completed within the time budget.

5. Timing measurements

Table S6. Timing measurement settings.

Experiment	Measurement
Table 9 CPU network timing	Core i9-14900HX, one thread, FP32; 2 and 3 bits; batches 1, 16, 64; initial QPP output; median of the three model-specific median times, with seven measurement blocks per model after warm-up.
Table 9 complete GPU inference	RTX 4070 Laptop; five fixed-variance 2-bit checkpoints; 16 inputs per checkpoint; 14 calls per input after warm-up. Includes CPU-to-GPU input transfer, network, QPP, TASL and GPU-to-CPU output transfer.
Scenario-count timing	Sr = 32, 64, 128, 256; one model (seed 1101), four inputs, and seven timed calls per input and scenario count after three warm-up calls. Batch size 1; inputs stored on the GPU. Timing covers network inference and phase refinement, excluding data transfer and CUDA graph preparation.

GPU results report the mean and sample standard deviation of checkpoint-specific medians, unless the table explicitly reports the single-checkpoint median. Batch times are divided by batch size to obtain milliseconds per sample. Loading model files and preparing the CUDA graphs occur before the timed calls.

6. Scenario-count results

Table S7. SE and computation time by scenario count.

Sr	Mean SE	Conditional lower-tail SE	Median time (ms)
32	6.092512	6.091579	6.7101
64	6.088092	6.089788	6.5315
128	6.082857	6.087113	6.6950
256	6.091471	6.092532	6.7790

SE is expressed in bit/s/Hz. The sample sizes for SE evaluation and timing are specified in Tables S5 and S6, respectively. Each reported time is the median of 28 calls for that scenario count.

7. Running the experiment code

In Dataset_S1/code, install the dependencies once, then start the main program:

```
python -m pip install -r requirements.txt
python main.py
```

Enter an experiment number to run it. The program loads the required settings and supplied data automatically and saves each run in a new folder under Dataset_S1/results.

Table S8. Experiments available from the main program.

Option	Experiment	Option	Experiment
1	Method comparison (supplied models)	6	Paired statistics
2	CRTQ-BF training	7	Remote estimation (supplied SE sequences)
3	CARAR replacement / matched budgets	8	Direct-path channel SE (supplied beams)
4	Deployment scenario-count sensitivity	9	Perfect-CSI numerical references
5	Deployment scenario-count GPU timing	10	Phase-resolution sweep (1-6 bits)
